# A hypomorphic *cystathionine ß-synthase* gene contributes to cavefish eye loss by disrupting optic vasculature

**DOI:** 10.1038/s41467-020-16497-x

**Published:** 2020-06-02

**Authors:** Li Ma, Aniket V. Gore, Daniel Castranova, Janet Shi, Mandy Ng, Kelly A. Tomins, Corine M. van der Weele, Brant M. Weinstein, William R. Jeffery

**Affiliations:** 1 Department of Biology, University of Maryland, College Park, MD USA; 2 Division of Developmental Biology, Eunice Kennedy Shriver National Institute of Child Health and Human Development, NIH, Bethesda, MD USA; 3 Department of Genetics, Stanford University, School of Medicine, Stanford, CA USA

**Keywords:** Developmental biology, Evolutionary developmental biology

## Abstract

Vestigial structures are key indicators of evolutionary descent, but the mechanisms underlying their development are poorly understood. This study examines vestigial eye formation in the teleost *Astyanax mexicanus*, which consists of a sighted surface-dwelling morph and multiple populations of blind cave morphs. Cavefish embryos initially develop eyes, but they subsequently degenerate and become vestigial structures embedded in the head. The mutated genes involved in cavefish vestigial eye formation have not been characterized. Here we identify *cystathionine ß-synthase a* (*cbsa*), which encodes the key enzyme of the transsulfuration pathway, as one of the mutated genes responsible for eye degeneration in multiple cavefish populations. The inactivation of *cbsa* affects eye development by increasing the transsulfuration intermediate homocysteine and inducing defects in optic vasculature, which result in aneurysms and eye hemorrhages. Our findings suggest that localized modifications in the circulatory system may have contributed to the evolution of vestigial eyes in cavefish.

## Abstract (web summary)

The teleost *Astyanax mexicanus* has sighted morphs living in surface rivers and various blind morphs living in caves. Here, the authors suggest that loss of eyes in cave morphs is linked to mutations in the *cystathionine ß-synthase a* (*cbsa*) gene, which cause eye degeneration by disrupting function of the optic circulatory system.

## Introduction

Vestigial structures, such as the reduced wings of flightless birds, the hind limb remnants of pythons, and the degenerate eyes of blind cavefish (CF), were key factors in Darwin’s recognition of descent with modification during evolution. However, the genetic and physiological mechanisms responsible for the development of rudimentary structures are still poorly understood.

In the teleost *Astyanax mexicanus*, blind CF has been derived repeatedly from sighted surface fish (SF) ancestors^1–3^. The loss of eyes in *A. mexicanus* CF involves initial optic development followed by subsequent degeneration^1,2^. Eye primordia with a lens and retina are formed in CF embryos, but the lens undergoes massive apoptosis, the retina becomes apoptotic and disorganized, and eye growth is eventually arrested during larval development^3–5^. Accordingly, the rate of optic growth fails to increase, and the small non-functional eyes are overgrown by skin and connective tissue as CF larvae develop into adults. About 30 distinct CF populations have evolved in Mexican caves, and several of these CF populations have evolved vestigial eye phenotypes independently^6,7^. Similar eye reduction or loss occurs in many cave-dwelling species^8^ and animals adapted to other dark habitats^9^.

The existence of interfertile morphs in *A. mexicanus* has allowed eye degeneration to be studied by genetic methods^2^. These studies have shown that eye loss in the Pachón CF (PA-CF) population is controlled by multiple genetic factors^10–12^. Furthermore, genetic complementation shows that some of the factors involved in eye loss are the same and others are unique in different CF populations^13^. In addition to genetic changes, epigenetic events may also have contributed to the evolution of eye loss in CF^14^. Quantitative trait locus (QTL) analysis revealed about 15 non-overlapping genomic regions that are responsible for lens and eye reduction in PA-CF^10–12,15^. The alignment of eye QTL with the sequenced PA-CF genome has suggested many candidates for genes controlling vestigial eye formation^15^. However, the identities of the genes and mutations within these QTL intervals have not been established.

Here we identify *cystathionine ß-synthase a* (*cbsa*), which encodes the key enzyme in the transsulfuration pathway, as one of the inactivated genes responsible for eye degeneration in multiple *A. mexicanus* CF populations. Studies of the hypomorphic *cbsa* phenotype revealed a mechanism for arresting eye growth based on the accumulation of the transsulfuration intermediate homocysteine (hCys) and disruption of optic vasculature. Our findings suggest that interference with circulatory system functions by the hypomorphic *cbsa* gene may have a crucial role in the evolution of CF eye degeneration.

## Results

### The *cbsa* gene as a candidate for CF eye loss

**Fig. 1:**
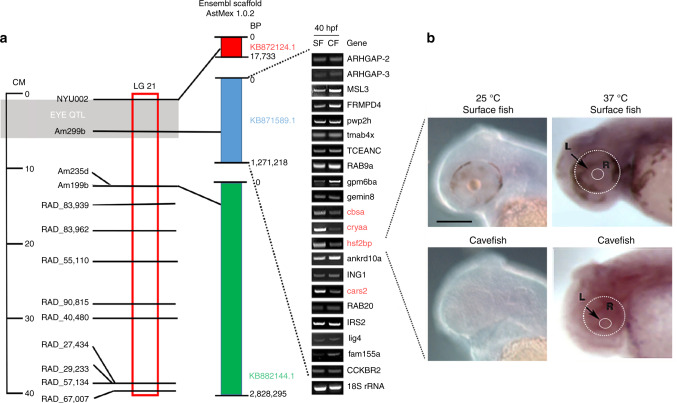
Gene expression screen of PA-CF genomic scaffold KB871589.1. **a** Location of an eye QTL on *A. mexicanus* linkage group 21 (left), alignment to nearby scaffolds of the draft PA-CF (AstMex1.0.2) genome sequence based on the position of markers (center), and qualitative RT-PCR analysis of the expression of 21 predicted scaffold genes at 40 hpf (right). Expression of genes in SF and PA-CF are shown from top to bottom according to their 5’ to 3’ order on the scaffold. CM centimorgans, LG linkage group. See Supplementary Table 5 for PA-CF gene IDs. Genes downregulated in PA-CF compared to SF are shown in red. Information based on at least three biological replicates. **b** In situ hybridization showing *hsf2bp* expression in 40 hpf SF and PA-CF embryos at 25 °C (left) and after treatment at 37 °C for 1 h (right). Dashed lines encircle the eyes and lenses in the right frames. *n* = 20 for all frames. L lens, R retina. Scale bar: 200 μm; same magnification in all frames. Source data are provided in source data file.

To identify a mutated gene responsible for CF eye loss, we examined *A*. *mexicanus* Ensembl Scaffold KB871589.1, which contains the peak marker of an eye size QTL located between the *hsf2bp* and *ankrd10a* genes (Fig. 1a)^11–13,15^. According to the Ensembl AstMex 1.0.2 genome assembly, this scaffold contains 21 predicted protein-coding genes. We surveyed these genes for expression differences in SF and PA-CF by qualitative reverse transcriptase polymerase chain reaction (RT-PCR) at 40 hours post-fertilization (hpf) (Fig. 1a; Supplementary Table 5), when changes associated with eye degeneration first appear in CF^1–5^. Since CF eye degeneration is a recessive trait^2–5^, we focused on the genes downregulated in PA-CF relative to SF and identified *cars2*, *αA-crystallin* (*cryaa*), *hsf2bp*, and *cystathionine ß-synthase a* (*cbsa*) (Fig. 1a). The *cars*2 gene, which encodes a mitochondrial aminoacyl-tRNA synthetase^16^ and is likely to function and be expressed ubiquitously, and *cryaa*, which was previously shown to be under *trans*-acting regulation during CF development^17^, were excluded from further study. Of the two remaining genes, the *hsf2bp* gene, which encodes a heat shock transcription factor 2 binding protein^18^, is located in close proximity to the peak QTL marker. We used in situ hybridization to determine the pattern of *hsf2bp* expression during normal culture at 25 °C and following a 1-h 37 °C heat shock (Fig. 1b). The results indicated that *hsf2bp* expression was ubiquitous in SF and PA-CF development, and although expression was increased by the heat shock, the increase was not particularly strong in the eyes (Fig. 1b). Thus our attention was focused on *cbsa*, which is expressed in the developing eyes in *A. mexicanus* and was predicted as a candidate for congenital anomaly in CF eye loss by Ingenuity Pathway Analysis of gene expression data^15^.

**Fig. 2:**
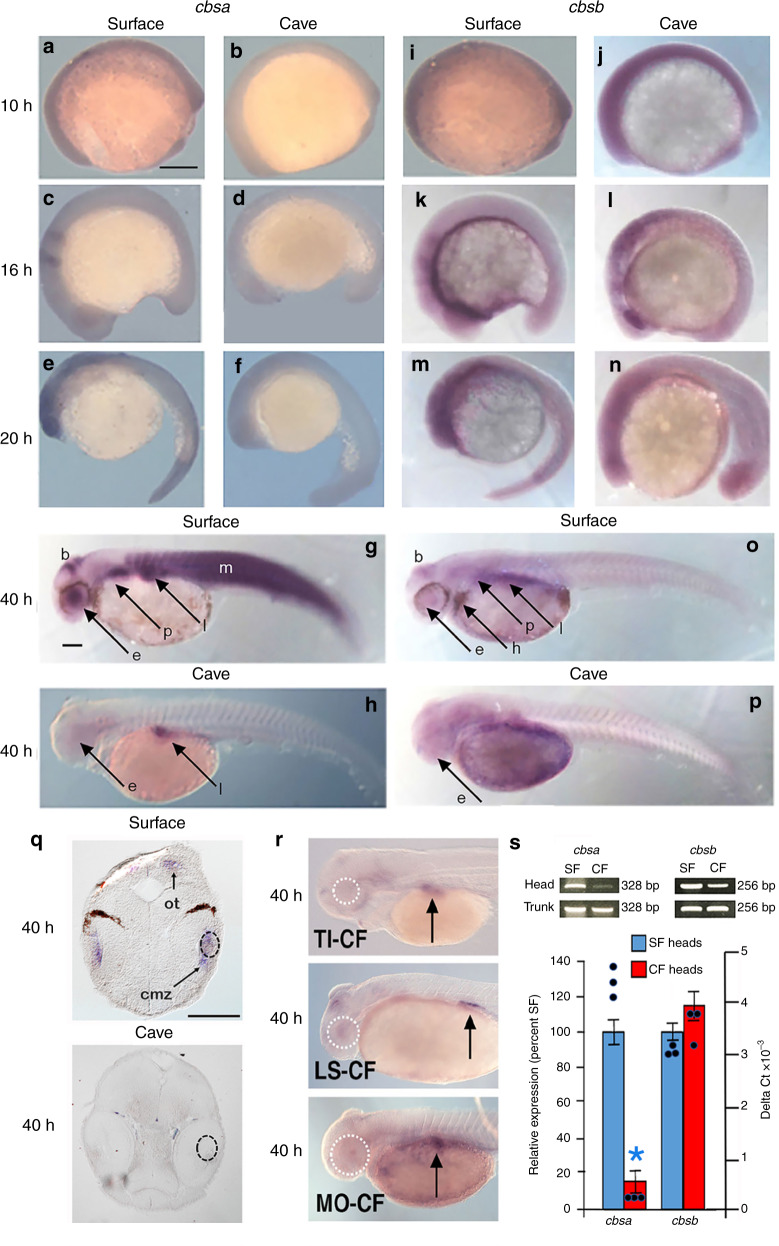
Expression of *cbsa* and *cbsb* genes during *Astyanax* development. **a**–**p** In situ hybridization showing *cbsa* (**a**–**h**) and *cbsb* (**i**–**p**) expression in SF and PA-CF embryos and larvae. b brain, e eyes, h heart p pectoral fin buds, l liver, m myotomes. *n* = 500 and at least 10 samples in each frame. **q** Sections through the heads of SF and PA-CF in situ hybridized larvae shown in **g** and **h**, respectively. ot optic tectum, cmz ciliary marginal zone of retina. Dotted lines: lenses. Top of frame is dorsal. **r** In situ hybridization showing *cbsa* expression in TI-CF, LS-CF, and MO-CF larvae at 40 hpf. Arrows: liver. Scale bar in **a** is 200 μm; magnification is the same in **a**–**f** and **i**–**n**. *n* = 140, 220, 240, 24, and 29 from top to bottom. Scale bar in **g** is 250 μm; magnification is the same in **g**, **h**, **o**, **p**, **r**. Scale bar in **q** is 300 µm; magnification is the same in both frames. **s** Qualitative (top) and quantitative RT-PCR comparing the levels of *cbsa* and *cbsb* expression in PA-CF heads and trunks. Graph: Bars are means and SEM of CF ΔΔCt values converted to percentage of SF ΔΔCt values (left *y* axis). Data points are ΔCt values (right *y* axis). Asterisk: significance calculated from ΔCt values (*n* = 3) by unpaired Student’s *t* test (*p* = 0.00). Source data are provided in source data file.

There are two *cbs* paralogs in *Astyanax*: *cbsa* and *cbsb*^15^. We examined the expression of the paralogous *cbs* genes during SF and PA-CF development by in situ hybridization with gene-specific probes (Fig. 2a–q). At early stages of embryonic development, the patterns of *cbsa* and *cbsb* expression were similar in SF and PA-CF, including in the developing eye primordia. However, *cbsa* expression was weaker in PA-CF than in SF (Fig. 2a–f), and *cbsb* expression was generally stronger than *cbsa* in PA-CF (Fig. 2a–f, i–n). By 40 h of development, *cbsa* and *cbsb* were expressed in many developing organs (Fig. 2g, h, o, p). The expression of *cbsa* was strong in the SF head, including the eyes and brain, and in the heart, pectoral fin buds, liver, and myotomes (Fig. 2g), whereas in PA-CF *cbsa* expression was downregulated in all of these organs except the liver (Fig. 2h). Sections of SF larval heads at 40 hpf showed *cbsa* expression in the lens, the ciliary marginal zone of the retina, and the optic tectum, whereas no expression was apparent in these regions in CF (Fig. 2q). The expression of *cbsb* was similar in most of these locations in SF and PA-CF at 40 hpf, with the notable exception of the head and myotomes, where *cbsb* expression was reduced or undetectable in both PA-CF and SF (Fig. 2o, p). The pattern of *cbsa* and *cbsb* expression in *Astyanax* SF resembled that reported in zebrafish^19^. The results suggest that *cbsa* and *cbsb* show mostly overlapping expression during SF and PA-CF development, except for the PA-CF eyes, brain, and myotomes in which neither of the *cbs* paralogs was strongly expressed. Thus *cbsb* expression could compensate for downregulated *cbsa* expression in CF larvae everywhere except for the eyes, brain, and myotomes. The in situ hybridization results were confirmed by comparing *cbsa* and *cbsb* expression in isolated larval heads and trunks by qualitative and quantitative RT-PCR, which showed that *cbsa*, but not *cbsb*, expression was downregulated in PA-CF larval heads at 40 hpf (Fig. 2s). The finding of no changes in *cbsa* levels between CF and SF trunks in qualitative RT-PCR experiments, despite strong downregulation in the myotomes by in situ hybridization, can be explained by compensating upregulation in the liver (Fig. 2h).

Eye loss is thought to have evolved independently at least twice in different *A. mexicanus* CF populations^1,2,13^. Therefore, we conducted in situ hybridization to determine whether there was also a change in *cbsa* expression in other CF populations and found that Tinaja CF (TI-CF), Los Sabinos CF (LS-CF), and Molino CF (MO-CF) had patterns of *cbsa* downregulation similar to PA-CF, including strong downregulation in the eyes (Fig. 2r). These results establish *cbsa* as a candidate for controlling eye loss in multiple CF populations.

### CF *cbsa* gene contains hypomorphic mutations

**Fig. 3:**
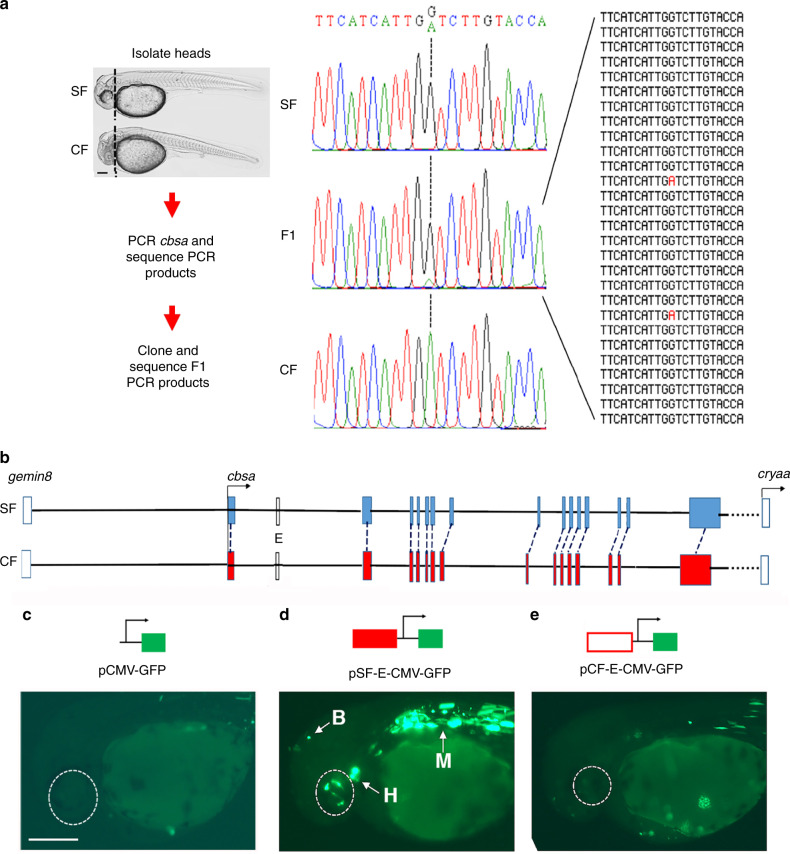
Structure and *cis*-regulation of the *Astyanax cbsa* gene. **a** F1 hybrid test. Left: summary of procedure beginning with separation of SF, F1 hybrid, and CF larvae into heads and trunks. Scale bar: 250 µm. Middle: sequenced *cbsa* RT-PCR products from SF, F1 hybrid, and PA-CF heads showing SF and PA-CF *cbsa* marker SNPs (A and G nucleotides, respectively). Right: marker region sequences of 28 independently cloned RT-PCR products from F1 hybrid heads showing the PA-CF allele marker in red. **b** Maps of re-sequenced SF and PA-CF *cbsa* loci showing intron–exon organization (colored boxes) and the predicted enhancer region E. Un-colored boxes: flanking *gemin8* and *cryaa* loci. **c**–**e** Expression of CMV-GFP constructs in SF at 40 hpf. **c** Control pCMV-GFP construct did not drive GFP expression. (*n* = 49). **d** SF *cbsa* enhancer E containing pCMV-GFP construct drove GFP expression in eyes (enclosed by dashed lines), heart (H), brain (B), and myotomes (M). (*n* = 22). **e** PA-CF enhancer E containing pCMV-GFP construct did not drive GFP expression. (*n* = 43). Dotted lines indicate eyes. Scale bar in **c** is 250 µm; magnification is the same in **c**–**e**. Source data are provided in source data file.

We next conducted F1 hybrid tests to determine whether downregulation of the *cbsa* gene is caused by *cis*- or *trans*-acting regulation in PA-CF^15,20^. In these experiments, SF females were crossed with PA-CF males to generate F1 hybrids, RNA was extracted from the heads of the F1 hybrid and parental larvae at 40 hpf, and a part of the *cbsa*-coding region containing a single-nucleotide polymorphism marker, which distinguished the SF and CF *cbsa* alleles, was amplified by PCR, cloned, and sequenced (Fig. 3a). The results showed that expression of the PA-CF *cbsa* allele was not increased in the F1 hybrid background, as expected under *trans*-acting control, thus implicating *cis*-regulation as the cause of *cbsa* downregulation.

To identify potential *cis*-regulating mutations, we sequenced the SF and PA-CF *cbsa* loci and immediate flanking regions. The results showed that the SF and CF *cbsa* genes consist of 15 exons (Fig. 3b). We used rapid amplification of cDNA ends (RACE) to identify the SF and CF *cbsa* transcripts and surveyed them for possible sequence differences. The 5’ non-coding regions of CF and SF *cbsa* mRNAs, which correspond to the first non-coding exon and the first few bases of the second exon, showed only two nucleotide differences. In addition, the CF *cbsa* coding region has no internal stop codons, suggesting that it encodes a complete cystathionine ß-synthase A (CBSA) protein (Supplementary Fig. 1a). The deduced PA-CF and SF CBSA proteins are 99% identical, differing by only five amino acids (Supplementary Fig. 1b). Only one of the divergent amino acids (G 257) is conserved in other known vertebrate CBS proteins. Although it is possible that PA-CF CBSA is functionally different from SF CBSA because it is missing a run of two amino acids (G257-K258; Supplementary Fig. 1c) that are present in other teleost CBS proteins, this difference would not explain the hypomorphic activity of the PA-CF *cbsa* gene.

Outside the protein-coding regions, the 7 kb non-coding region located between *cbsa* and *gemin8*, which shows 99% nucleotide identity between PA-CF and SF, has only a few substitutions, while the 5 kb non-coding region located between *cbsa* and *cryaa* is also highly conserved in SF and PA-CF^17^. In addition, *cbsa* introns are also highly conserved in PA-CF, except for the following: a run of 4 indels in a 500-bp region of intron 1, a 323-bp deletion in intron 6, and a 120-bp insertion in intron 8 (Supplementary Table 1; Supplementary Fig. 2). To explore the possibility that these introns contain potential *cis*-regulatory changes, we amplified and compared their sequences in the PA-CF, TI-CF, LS-CF, MO-CF, Chica CF (CH-CF), and Jineo CF (JI-CF) *cbsa* genes. Genetic studies have shown that eye loss evolved independently at least twice in these CF populations^2,11,13^, and our in situ hybridization data indicated that *cbsa* is downregulated in PA-CF, TI-CF, LS-CF, and MO-CF eyes (Fig. 2). We found that the indels in introns 6 and 8 were present in some, but not all, of these CF populations (Supplementary Table 1), suggesting that they are unlikely to include changes generally responsible for *cbsa* downregulation. In contrast, indels were found at the same position in intron 1 of all six CF populations (Supplementary Fig. 2), suggesting that this region is a candidate for containing *cis*-acting mutations.

We next searched for enhancers in the genomic region between the 3’ termini of the *gemin8* and the *cbsa* genes using iEnhancer-2L^21^. A putative 282-bp enhancer (E region, Fig. 3b) was predicted in *cbsa* intron 1, which overlapped the indels described above in all six CF populations. To test for regulatory function, we made DNA constructs in which a 354-bp genomic DNA fragment containing the E region was fused to a cytomegalovirus (CMV) promoter and green fluorescent protein (GFP) reporter gene, injected these constructs into SF eggs, and determined reporter activity at 40 hpf by GFP fluorescence (Fig. 3c–e). The CMV promoter alone drove modest GFP expression in SF myotomes, but no expression in the eyes or brain (Fig. 3c). In contrast, the pSF-E-CMV-GFP construct containing the SF *cbsa* E region drove strong GFP expression in the myotomes, eyes, heart, and the dorsal brain of 40 hpf SF embryos but not in the liver or other locations of normal *cbsa* expression (Fig. 3d). Thus the E region contains a tissue-specific enhancer for *cbsa* expression. Contrastingly, the pCF-E-CMV-GFP construct containing the E region of PA-CF *cbsa* drove very weak or no reporter expression in SF larval eyes, heart, and brain (Fig. 3e). These results suggest that *cis*-inactivating mutations in an enhancer located in the first intron are responsible for downregulation of the *cbsa* gene in PA-CF. However, we cannot exclude the possibility that other *cis*-acting changes, within or beyond the sequenced regions, may also contribute to *cbsa* downregulation. The indel regions in E contain many predicted transcription factor-binding sites that could promote restricted expression in the head and mediate changes in *cbsa* expression in CF and SF (Supplementary Fig. 3). These results (and functional data below) suggest that *cbsa* could be one of the mutated genes responsible for CF eye degeneration.

### CF show homocystinuria-like features

**Fig. 4:**
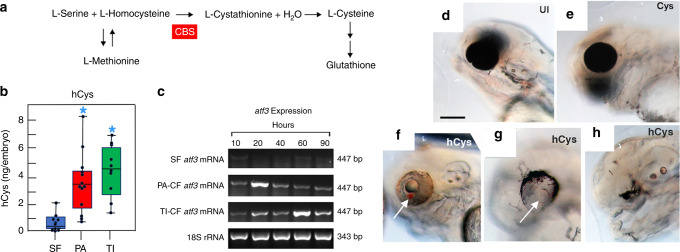
Homocysteine elevation and effects on eye development. **a** The transsulfuration pathway showing one of the reactions catalyzed by the CBS enzyme. **b** ELISA determination of hCys levels in PA-CF and TI-CF relative to SF at 40 hpf. Box plots with superimposed data points of 5–7 biological replicates. Graph represents box and whiskers min to max. Asterisks: *p* = 0.001 and 0.003 from left to right. Significance was determined by unpaired Student’s *t* test. **c** Expression of *atf3* during PA-CF, TI-CF, and SF development determined by qualitative RT-PCR. Three biological replicates with similar results. **d**–**h** Effects on eye development at 4 dpf after injection of 5 mM hCys into SF eggs (**f**). **d**, **e** Normal eyes in un-injected (UI) SF eggs (**d**) and SF eggs injected with 5 mM cysteine (Cys) (**e**). **f**–**h** Abnormal eyes with a hemorrhage (arrow) (**f**) or with a ventrally displaced lens (arrow) (**g**), and complete suppression of eye formation (**h**) in SF eggs injected with 5 mM hCys. SF in **f** was treated with PTU to visualize hemorrhage. Scale bar: 200 μm; same magnification in **d**–**h**. *n* values: see Supplementary Table 2. Source data are provided in source data file.

The *cbsa* gene encodes CBS, the limiting enzyme of the transsulfuration pathway, which converts hCys to cystathionine, a precursor of cysteine and glutathione^22^ (Fig. 4a). Mutations in the human *cbs* gene are the major cause of homocystinuria, a disorder in methionine metabolism resulting in toxic hCys accumulation and eye abnormalities^23,24^. In fact, the small eyes of CF larvae resemble the eyes of some homocystinuria patients in showing an “off-center” lens^2–5^. To test the possibility that CF show homocystinuria-like features, we compared hCys levels by enzyme-linked immunosorbent assay (ELISA) quantification and expression of *activating transcription factor 3* (*atf3*), a major hCys-responsive gene^25^, in CF and SF larvae. The results showed increases of hCys levels in PA-CF and TI-CF larvae at 40 hpf (Fig. 4b) and upregulation of the *atf3* gene in PA-CF and TI-CF relative to SF from about 1 to 4 days post-fertilization (dpf) (Fig. 4c), indicating that hCys accumulates in CF. To examine the developmental effects of increased hCys, we injected hCys into SF eggs and examined eye morphology at 40 hpf. The results showed that about 23% of the larvae that developed from SF eggs injected with hCys showed defects in eye formation, including reduced eye size, displacement of the lens to the ventral side of the retina, or complete eye loss (Fig. 4d–h; Supplementary Table 2), and in some cases hemorrhages occurred in or near the eyes (Fig. 4f; and see below). In contrast, only about 4% of control larvae that developed from eggs injected with cysteine showed effects on eye development (Fig. 4a, e; Supplementary Table 2), and no eye hemorrhages were seen in these embryos. These results indicate that CF exhibit homocystinuria-like features that could impact eye development.

### Defects in CF optic vasculature

**Fig. 5:**
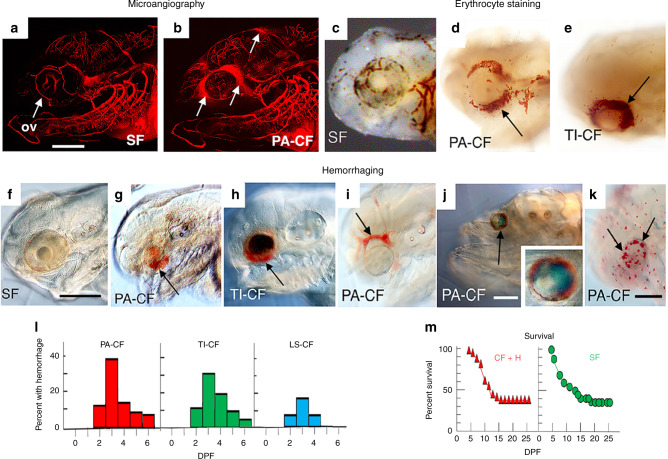
Defective optic vasculature in cavefish. **a**, **b** SF (**a**) and PA-CF (**b**) at 5 days post-fertilization (dpf) showing leaky vasculature around the PA-CF eye (arrows). ov optic vasculature. Each microangiography assay was conducted with three biological replicates. **c**–**e** SF (**c**), PA-CF (**d**), and TI-CF (**e**) showing stained erythrocytes (arrows in **d**, **e**) pooled around CF eyes at 2.5 dpf. *n* = 24 for SF and 36 for PA-CF. **f**–**h** SF (**f**), PA-CF (**g**), and TI-CF (**h**) at 4 dpf showing hemorrhages (arrows in **g**, **h**) in CF eyes. **i** Aneurysm (arrow) in PA-CF optic vasculature at 4 dpf. **j** PA-CF showing eye hemorrhage (arrow) at 14 dpf (inset, 2.5 magnification of eye area). **k** Neutral red staining of PA-CF at 7 dpf showing macrophages (arrows) in degenerating eyes (*n* = 36 larvae). Scale bars: **a** 300 μm (same magnification in **a**, **b**), **f** 200 μm (same magnification in **c–i**), **j**, **k** 150 μm. **l** The percentage of PA-CF (*n* = 2101), TI-CF (*n* = 268), and LS-CF (*n* = 121) with hemorrhages during larval development. **m** Survival of SF (right) (*n* = 100) and PA-CF (*n* = 46) with hemorrhages (left) during larval development. Source data are provided in source data file.

High hCys levels are known to affect cardiovascular function, increasing the risk of blood clots, aneurysms, and hemorrhagic stroke in homocystinuria patients and *Cbs*^+/−^ mice^26,27^. Therefore, we examined optic vasculature in CF by microangiography, blood cell staining, and eye imaging (Fig. 5). The results revealed leaky optic and brain vasculature, erythrocyte pooling and aneurysms around the eyes, and eye hemorrhages (Fig. 5a–j), which were observed in up to 20–40% PA-CF, TI-CF, and LS-CF larvae (Fig. 5l) but not in >1000 SF larvae (Fig. 5f). The eye hemorrhages were generally unilateral, ranging from small foci on the dorsal side of the eye to large areas of the orbit and in most cases persisted for only about 3–4 days (Fig. 5l). During this period, the degenerating CF eyes were invaded by phagocytic macrophages, probably to remove leaked blood cells (Fig. 5k). Survival studies showed that PA-CF larvae with eye hemorrhages had similar viability to SF larvae (Fig. 5m), showing that defective optic vasculature does not affect CF viability. The results indicate that defects in optic vasculature accompany CF eye degeneration.

### Role of *cbs* genes in eye and optic vasculature development

**Fig. 6:**
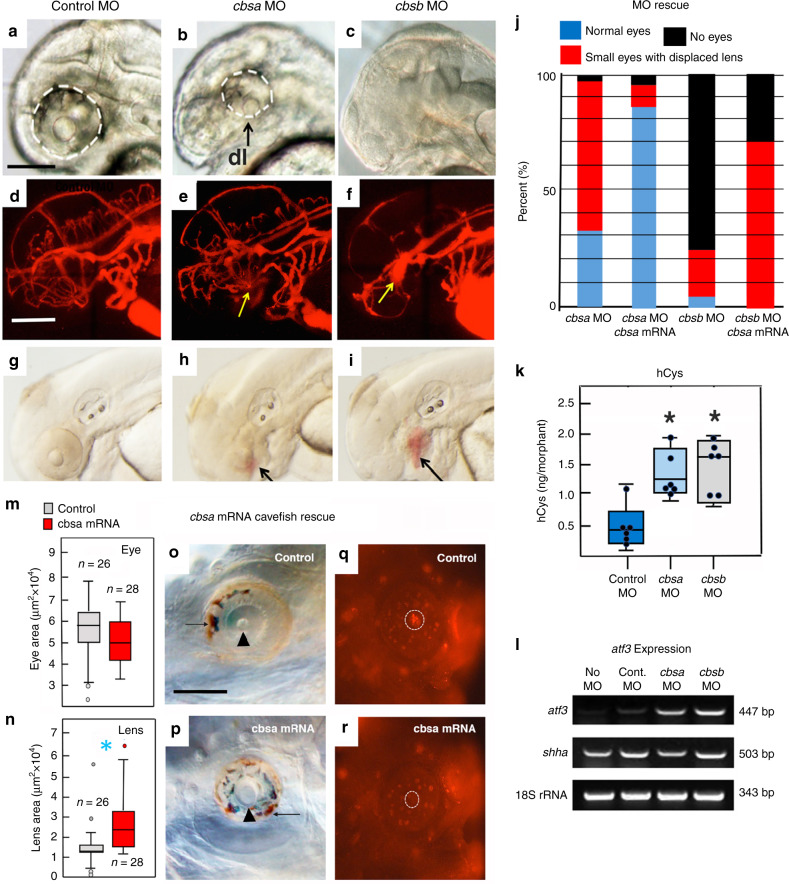
Effects of *cbs* gene knockdown and rescue by *cbsa* overexpression. **a**–**i** Morpholino (MO) knockdown of *cbsa* and *cbsb* genes. Effects of control (**a**, **d**, **g**), *cbsa* (**b**, **e**, **h**), and *cbsb* (**c**, **f**, **i**) MOs on eye development (**a**–**c**), optic vasculature integrity determined by microangiography (**d**–**f**), and optic hemorrhage formation (**g**–**i**) in SF at 40 hpf. Each assay was conducted with three biological replicates. Dashed lines: eyes. Yellow arrows: leaky vasculature. dl displaced lens, h hemorrhages. Scale bar in **a** is 200 µm; magnification is the same in **a**–**i**. **j** Partial reversal of *cbsa* MO- and *cbsb* MO-induced eye defects in SF morphants by injection of *cbsa* mRNA. Bar graphs show the percentage of normal and abnormal eye phenotypes. See Supplementary Table 4 for *n*. Significance by chi-square test. Chi^2^ is 129.9987; *p* < 0.001. **k**, **l** Effects of *cbs* MO knockdown on hCys levels (**k**) and *atf3* and *shha* gene expression (**l**) in SF at 40 hpf. **k** Box plots with superimposed data points of six biological replicates. Significance was determined by unpaired Student’s *t* test. (*p* = 0.006 and 0.003 from left to right). **m**–**r** Partial rescue of eye phenotypes by SF *cbsa* mRNA injection in PA-CF embryos. **m**, **n** Box plots with superimposed data points showing mean eye (**m**) and lens (**n**) sizes at 6.5 dpf in control (**o**) and mRNA injected PA-CF embryos (**p**). *n* is shown above or below boxes. Significance was determined by unpaired Student’s *t* test. (*p* = 0.003). **o**, **p** Eyes from **m** (**o**) and *n* (**p**) showing macrophages (arrows). Arrowhead: lens. **q**, **r** Lens apoptosis in control (**q**) but not in mRNA injected (**r**) PA-CF embryos. Dashed lines: lens. *n* = 22 for CF and 30 for SF. Asterisks: Significance was determined by unpaired Student’s *t* test. (*p* = 0.006). Scale bar in **o** is 350 µm; magnification is the same in **o**–**r**. Graphs represent box and whiskers min to max. Source data are provided in source data file.

To address the roles of *cbs* genes in eye and vasculature development, we knocked down *cbsa* and *cbsb* expression by injecting SF eggs with splice-inhibiting morpholinos (MOs) (Fig. 6a–l). To validate this approach, we amplified and sequenced PCR products from the MO-targeted regions of *cbs* morphants and obtained products of two different sizes: one with sequence corresponding to the processed *cbsa* exons 5 and 6 or *cbsb* exons 3 and 4, and the other with sequence including the unprocessed introns containing the expected translation stop sites (Supplementary Fig. 4a–f). The *cbsa* and *cbsb* MOs were specific for the corresponding genes, respectively (Supplementary Fig. 4a, b).

We next determined the effects of *cbsa* and *cbsb* MO knockdown on eye development. About 40% of *cbsa* morphants developed morphologically abnormal eyes: a small proportion of these completely lacked eyes and the remainder had reduced eyes compared to those injected with control MO, including ventrally displaced lenses with decreased expression of the lens marker gene *cryaa* (Fig. 6a, b; Supplementary Table 3; Supplementary Fig. 4g, h), which is similar to the CF eye phenotype^2–5^. About 82% of the *cbsb* morphants lacked eyes and *cryaa* expression, whereas the remainder had reduced eyes compared to controls and showed ventrally displaced lenses (Fig. 6a, c; Supplementary Table 3; Supplementary Fig. 4g, h). No other morphological changes were noted in these *cbs* morphants (Supplementary Fig. 4g). The control morphants showed about 97% normal optic phenotypes (Fig. 6a; Supplementary Table 3; Supplementary Fig. 4g, h). Co-injection of *cbsa* and *cbsb* MOs generated eye phenotypes resembling the single *cbsb* MO injections (Supplementary Table 3). We also tested whether the morphant eye phenotypes could be rescued by injection with SF *cbsa* mRNA. The results showed that about 85% of *cbsa* mRNA-injected *cbsa* morphants developed morphologically normal eyes, compared to about 30% with normal eyes in *cbsa* morphants that were not injected with *cbsa* mRNA (Fig. 6j; Supplementary Table 4). Furthermore, the proportion of *cbsa* mRNA-injected *cbsb* morphants lacking eyes was reduced to 30%, compared to 75% in *cbsb* morphants that were not injected with *cbsa* mRNA, although normal eyes were not obtained in the *cbsa* mRNA-injected *cbsb* morphants (Fig. 6j; Supplementary Table 4). These results further support the specificity of *cbs* MO effects.

To determine whether the CF eye degeneration phenotype is reversible, SF *cbsa* mRNA was injected into 1-cell PA-CF embryos and evaluated for eye morphology and lens apoptosis at 5.5 dpf (Fig. 6m–r). Although no increases in mean eye size were observed between the *cbsa* mRNA-injected and control larvae (Fig. 6m), the *cbsa* mRNA-injected larvae showed a significant increase in average lens size compared to controls (Fig. 6n). Importantly, lens apoptosis, a key feature of CF eye degeneration^2,4^, was substantially reduced or not observed in the *cbsa* mRNA-injected PA-CF larvae, whereas the control PA-CF larvae showed typical lens apoptosis, and no differences in retinal apoptosis were detected between *cbsa* mRNA-injected and control CF larvae (Fig. 6q, r). Other aspects of the eye degeneration phenotype, such as the formation of optic hemorrhages and the invasion of macrophages, were not altered by *cbsa* mRNA injection (Fig. 6o, p). The results indicate that *cbsa* overexpression can partially reverse CF eye degeneration.

**Fig. 7:**
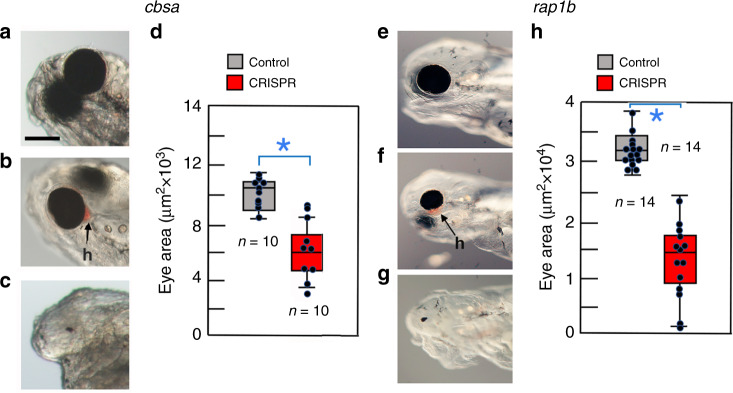
Effects of *cbsa* and *rap1b* gene editing on eye development. **a**–**c**, **e**–**g** Control (**a**, **e**) and CRISPR-Cas9-edited (**b**, **c**, **f**, **g**) larvae showing the range of eye phenotypes. **d**, **h** Box plots comparing mean eye size in control and CRISPR-edited larvae at 3.5 dpf. Graphs represent box and whiskers min to max. *n* is shown above or below each box. Error bars: SEM. Asterisks indicate significance in unpaired Student’s *t* test. (*p* = 0.000 in **d**, **h**). *n* is shown near box plots. Scale bar in **a** is 250 µm; magnification is the same in **a**–**c** and **e**–**g**. Source data are provided in source data file.

We also examined the effects of *cbs* MO knockdown on hCys accumulation and optic vasculature development. ELISA quantification showed that hCys levels were increased in *cbsa* and *cbsb* morphants compared to controls (Fig. 6k), and increased *atf3* expression, but not the control *sonic hedgehog a* (*shha*) gene, was also observed in *cbs* morphants (Fig. 6l). These results show that *cbs* knockdown increases hCys levels. To determine the effects on vasculature development, we conducted microangiography of *cbs* and control MO-injected larvae. The *cbs* morphants showed leaky vasculature around the developing eyes and in the brain (Fig. 6e, f), whereas vascular integrity was not compromised in control morphants (Fig. 6d). Moreover, imaging indicated that *cbsa* and *cbsb* morphants developed hemorrhages around the eyes (Fig. 6h, i), but hemorrhages were not observed in control morphants (Fig. 6g). These results demonstrate that the hCys and optic vasculature phenotypes of CF can be generated in SF by *cbs* gene knockdown. The effects of *cbsa* MO knockdown on SF eye development were confirmed by clustered regularly interspaced short palindromic repeats (CRISPR)-Cas9 editing of the *cbsa* gene (Fig. 7a–d; Supplementary Fig. 5a). The *cbsa* exon 2 was targeted by two guide RNAs, and the mosaic F0 larvae were scored for eye phenotypes. The *cbsa-*edited larvae showed eye abnormalities ranging from reduced eyes with hemorrhages to completely absent eyes (Fig. 7a–d). In summary, the MO knockdown and CRISPR-Cas9 editing results show that blocking *cbs* gene expression increases hCys levels, induces defects in optic vasculature, and generates abnormal eye morphology, suggesting a role for the circulatory system in CF eye degeneration.

### Inhibition of circulatory function affects eye growth

To confirm the role of the circulatory system in CF eye degeneration, we used CRISPR/Cas9 to edit the *rap1b* gene in SF and determined the effects on eye size and morphology in the F0 generation (Fig. 7e–h; Supplementary Fig. 5b). The *rap1b* gene is required in endothelial cells for vascular integrity and in mutant form shows cranial hemorrhages in zebrafish^28^, resembling the *Astyanax* CF and SF *cbs* knockdown phenotypes. The *rap1b*-edited and control larvae showed eyes of similar size at 2.5 dpf, but by 3.5 dpf eye size was much smaller in the CRISPR-edited larvae than in the control larvae, and in some cases eye hemorrhages were formed or eyes were missing (Fig. 7e–h). These results suggest that interference with circulatory system function in *rap1b* mutants can phenocopy the effects of hypomorphic *cbsa*, confirming the conclusion that defective optic vasculature is an important regulator of CF eye degeneration.

### Degenerating CF eyes exhibit hypoxia-related stress

**Fig. 8:**
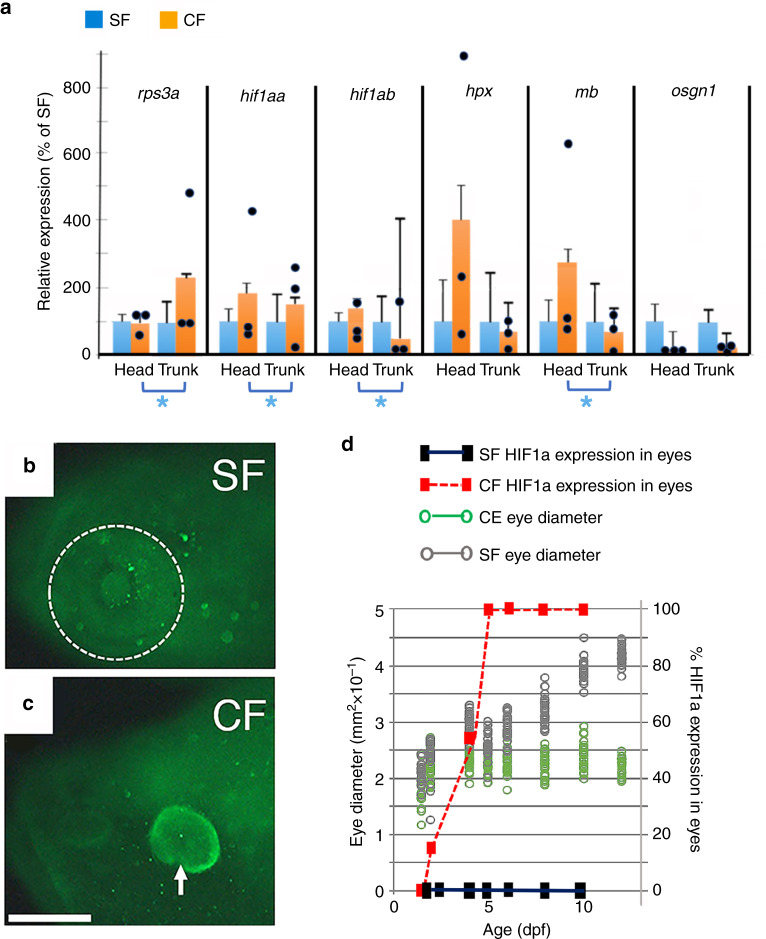
Hypoxia in cavefish. **a** Expression of hypoxia-sensitive genes by qPCR in isolated SF and PA-CF heads and trunks. Bars are means and SEM of CF 2^−ΔΔCt^ values converted to percentage of SF values in three biological replicates. Data points are ΔΔCt values (*n* = 3). Asterisks: significance calculated from ΔΔCt values by unpaired Student’s *t* test (*p* = 0.000, 0.002, 0.002, and 0.002 from left to right). **b**, **c** Determination of hypoxia-inducing factor 1α (HIF1a) expression in SF (**b**) and PA-CF (**c**) eyes at 7 dpf by immunostaining. Dotted line surrounds SF eye. Arrow indicates the margin of CF eye. SF: *n* = 16. PA-CF: *n* = 16. Scale bar: 150 μm; same magnification in **b** and **c**. **d** HIF1α expression in CF eyes determined by immunostaining compared to optic growth during SF and PA-CF development. *n* = 16 larvae for each time point. Source data are provided in source data file.

The discovery of defective optic vasculature opened the possibility that CF eyes may be subject to hypoxic stress. To explore this possibility, the *Astyanax* homologs of some of the genes responsive to hypoxia-related stress in zebrafish^29,30^ were identified, and their expression levels were compared in SF and PA-CF larvae that were separated into heads and trunks at 36 hpf (Fig. 8a, b). We selected the *hypoxia-inducible factor 1αa* (*hif1αa*) and *hif1αb*, which encode master regulators of hypoxia^31^ (but note that HIFα can also be induced by other types of stress); *hemopexin* (*hpx*), which encodes a major hemolysis-sensitive heme scavenger^32^; *myoglobin* (*mb*), which encodes an oxygen transporter in muscle cells^33^; and *oxidative stress growth inhibitor 1* (*osgn1*), which encodes an oxidative stress-response protein that regulates cell death, as examples of genes upregulated by hypoxia in zebrafish. We also selected the *rps3a* gene, encoding a ribosomal protein that is upregulated by hypoxic stress in the liver of another teleost^34^. The results showed that the *hif1αa*, *hif1αb*, and *mb* genes were upregulated in the head compared to the trunk of PA-CF larvae (Fig. 8a). Furthermore, *rps3a* was upregulated in the PA-CF trunk, where the liver is located, compared to the head (Fig. 8a). No significant differences were seen in the expression of these genes between the head and trunk of SF larvae (Fig. 8a). The results suggest that the expression of genes related to hypoxic stress are increased in CF heads. To investigate hypoxic stress in degenerating CF eyes, we stained CF and SF controls with an antibody against the master hypoxia-regulating factor HIF1α. Early in development, when both SF and PA-CF eyes increase in size, HIF1α was not detected in SF or PA-CF eyes, but as optic development proceeded and CF eye growth was arrested, HIF1α was seen in CF, but not in SF eyes, and HIF1α continued to be present through the subsequent eye degeneration (Fig. 8b–d). These results suggest that degenerating CF eyes show hallmarks of hypoxic stress.

### Optic vasculature disruption is independent of lens apoptosis

**Fig. 9:**
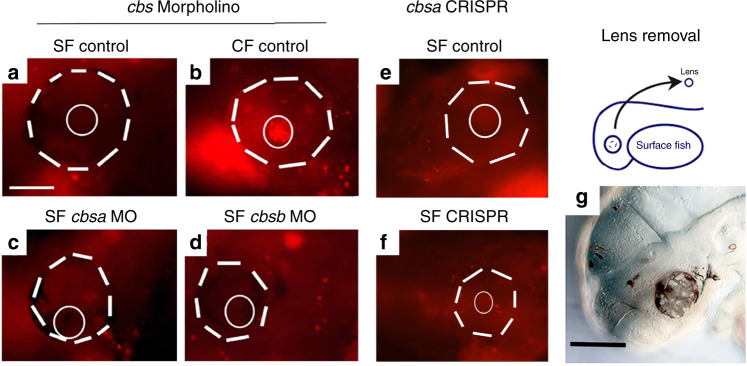
The expression of *cbs* genes and lens-associated phenotypes. **a**–**f** Apoptosis detected by Lysotracker staining in *cbs* morphant larvae and controls at 68 hpf. **a** SF control with no lens apoptosis (*n* = 14). **b** PA-CF control with lens apoptosis. (*n* = 26). *cbsa* (**c**) and *cbsb* (**d**) SF morphants with no lens apoptosis. (*n* = 22 and 14, respectively). **e**, **f** Apoptosis detected by Lysotracker staining in *cbsa* CRISPR-Cas9-edited SF larvae at 60 dpf: **e** SF control with no apoptosis (*N* = 24); **f** SF CRISPR-Cas9 with no lens apoptosis (*n* = 30). Dashed lines: outline of eyes. Complete lines: outline of lenses. **g** Lens removal from the optic cup on one side of SF larvae at 30 hpf (illustration of operation shown on top) did not induce optic hemorrhages at 4 dpf. (*n* = 23). Scale bar in **a**: 100 μm; Magnifications are the same in **a**–**f**. Source data are provided in source data file.

Previous studies showed that lens apoptosis is an early mediator of CF eye degeneration^2–5^. Because apoptosis and reduced lens size in CF could be partially rescued by overexpression of *cbsa* in CF (Fig. 6m–r), we conducted additional experiments to understand the relationship between CF lens dysfunction, *cbsa* downregulation, and optic vasculature defects. First, we assayed lens apoptosis in *cbs* morphants and CRISPR-Cas9-edited SF and CF control larvae at 40 hpf. Apoptotic cells were detected in the lens of CF controls (Fig. 9b) but not in the lens of SF controls (Fig. 9a) or in the lenses of *cbsa* and *cbsb* morphants or *cbsa* CRISPR-Cas9-edited larvae (Fig. 9c, d, f), showing that interference with *cbs* function does not promote lens apoptosis. Second, we removed a lens from eyes on one side of the SF larval head at 30 hpf and asked whether this operation induced the formation of optic hemorrhages later in development. The results showed that optic hemorrhages were not induced in SF larvae lacking a single lens (Fig. 9g). Together, these results suggest that lens dysfunction and hypomorphic *cbsa*-mediated optic vasculature defects are independent events in CF eye degeneration.

## Discussion

In the present study, we have identified *cbsa* as one of the genes responsible for eye degeneration in *A. mexicanus* CF. Furthermore, studies of the hypomorphic *cbsa* phenotype suggest a mechanism for eye degeneration based on the disruption of optic circulatory system function. Our work builds on previous studies that determined the multiplicity of genetic factors involved in CF eye loss^1,2,10^, identified QTL and corresponding genomic regions responsible for the regressive CF eye phenotype^11–13,15^, and tested candidate genes for mutations and roles in CF eye degeneration^17^. However, none of the mutated genes responsible for CF eye loss were identified.

Our study, along with the studies mentioned above, provides multiple lines of evidence suggesting that *cbsa* is one of the mutated genes responsible for CF eye degeneration. The *cbsa* gene is located near the peak marker of a PA-CF eye size QTL^11,15^, was recognized as a potential candidate for controlling CF eye loss by Ingenuity Pathway Analysis of gene expression data^15^, and is downregulated in eyes during larval development in four different CF populations: PA-CF, LS-CF, TI-CF, and MO-CF. Importantly, two or more of these CF populations are thought to have evolved the eyeless phenotype independently and share some of the same genetic factors involved in eye loss^13^. Our results suggest that *cbsa* may be one of the shared genes. We have also presented MO knockdown and CRISPR-Cas9 gene-editing results showing that the *cbsa* gene is required for SF eye development. When *cbsa* function was disrupted, eye formation was completely abolished or abnormal eyes with ventrally displaced lenses—a part of the typical CF larval eye phenotype^2–5^—were formed without affecting general larval morphology. Our results are consistent with the possibility that the hypomorphic *cbsa* gene is responsible for the phenotypic effects represented by an *Astyanax* eye QTL. However, more functional testing of other genes within the genomic interval will be needed to conclude that it is the only gene in this QTL that is mutated and involved in eye degeneration. Although the lens degeneration phenotype was partially reversed by overexpressing *cbsa* in CF embryos, there was no rescue of eye size or hemorrhage formation, indicating that *cbsa* does not have the power to reverse the entire CF eye degeneration phenotype. In this regard, it is important to note that *cbsa* is only one of many genetically^10–13,15^ or epigenetically^14^ modified genes that may regulate CF eye degeneration and that other genes remain to be discovered to fully understand this complex phenotype.

Our studies of the CF *cbsa* phenotype revealed that *A. mexicanus* CF show similarities to the human disease homocystinuria and mouse *Cbs* models of this disease with respect to eye abnormalities, elevated hCys levels, and deficiencies in the cardiovascular system^23–27^. We found approximately 3–4-fold increases in hCys in PA-CF and TI-CF larvae relative to SF larvae. Heterozygous *Cbs*^+/−^ mice show a 2-fold elevation of hCys levels, which results in alterations of retinal vasculature, and a 40-fold increase in hCys causes death in utero in *Cbs*^−/−^ mice^26,27,35^. The ability of CF to survive mild homocystinura-like effects and develop into healthily adults is likely due to compensation for *cbsa* downregulation by the paralogous *cbsb* gene. The *Astyanax cbsa* and *cbsb* genes do not appear to be co-expressed in the eyes, which is the likely reason that hypomorphic *cbsa* effects are restricted to these organs and do not engender a lethal response. We also observed *cbsa* expression in the SF optic tectum, as also reported for *cbs* genes in zebrafish^19^ and mouse^36^, and *cbsa* is downregulated in this region of the CF brain. The optic tecta are regressed coordinately with the eyes during CF evolution^37,38^. Therefore, mutated *cbsa* could promote the regression of CF eyes and optic tecta as a functional unit.

Mutations in *cbs* reduce life expectancy in homocystinuria patients and are responsible for cardiovascular defects in mouse models of homocystinuria^24–27^. We discovered defects in optic and cranial vasculature, including vascular leakages, eye hemorrhages, and aneurysms, during the critical period of CF eye degeneration. In our study, these defects were only seen after SF eggs were injected with hCys, a *cbs* paralog was knocked down by MO injection, or *cbsa* was edited by CRISPR-Cas9, implying that normal *cbs* expression is required for vasculature development and/or function in *A. mexicanus*.

**Fig. 10:**
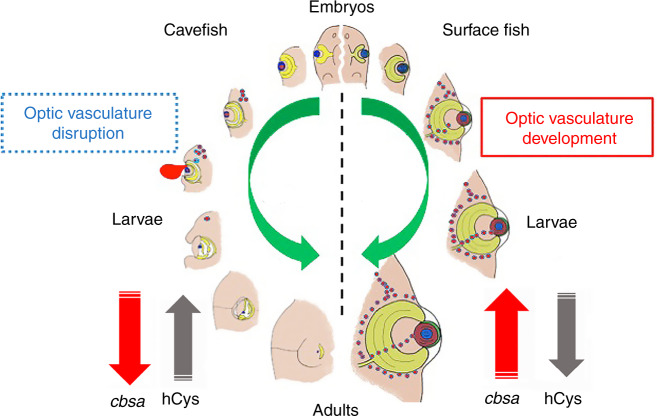
A model for cavefish eye degeneration. The model illustrates changes in *cbsa* expression (red upward pointing arrows indicate normal expression in surface fish; red downward pointing arrows indicate downregulated expression in cavefish) resulting in hCys elevation (black downward pointing arrows show normal hCys titers in surface fish; black upward pointing arrows indicate elevated titers in cavefish) and the induction of defects in optic vasculature (red dots surrounding and within developing eyes) that can result in hemorrhages in cavefish (red ellipse; left). Green arrows show trajectories of eye degeneration in cavefish larvae (left) or eye development in surface fish larvae (right).

Our results suggest that circulatory system dysfunction triggered by *cbsa* downregulation is important for inhibiting eye growth in *Astyanax*. We substantiated this conclusion by showing that eye growth is arrested by CRISPR-Cas9 editing of the *rap1b* gene in SF, which is required for vascular integrity and in mutant form can induce eye hemorrhages during larval development. The reliance of eyes on oxygen, nutrients, or humoral factors provided by blood flow^39,40^ would explain why CF form eyes early in embryogenesis and eye regression begins later during larval development. We also showed that hypoxia gene markers, including the master hypoxia regulator HIF1α, are increased in CF heads or eyes. Therefore, a model is proposed in which deficiencies in oxygen or other factors normally provided by the optic circulatory system are responsible for CF eye regression (Fig. 10). Importantly, we infer that defective circulation, rather than the effects of hemorrhages or aneurysms themselves, is the critical factor induced by the hypomorphic *cbsa* locus. Interference with eye growth by the induction of localized hypoxic stress may confer an evolutionary benefit for CF by eliminating the high energetic cost of maintaining eyes, as vision is useless in the dark cave environment^41,42^.

Previous studies showed that lens dysfunction is involved in the early stages of CF eye degeneration but also recognized that additional factors must be involved^4,10-13^. Although we have shown here that lens size and apoptosis can be rescued in CF by *cbsa* overexpression, lens apoptosis was not induced in *cbs* morphants or *cbsa* CRISPR-Cas9-edited larvae. Therefore, our results suggest that defects in the CF lens and optic vasculature are governed by different genes and mechanisms that cooperate to produce the complex CF eye regression trait. It will be interesting to determine whether the other genes contribute to the defective optic vasculature phenotype, the lens dysfunction phenotype, both of these phenotypes, or reveal additional changes involved in vestigial eye formation.

## Methods

### Husbandry and general experimental procedures

*A. mexicanus* SF, PA-CF, TI-CF, LS-CF, JI-CF, CH-CF, and MO-CF were obtained from stocks in the Jeffery laboratory. Fish were raised in the laboratory at 25 °C on a 14-h light and 10-h dark photoperiod, and spawning was induced by a temperature increase^43^. Embryos and larvae were raised at 23–25 °C. Some SF embryos were cultured with 400 μM phenylthiourea (Sigma-Aldrich, St. Louis, MO) to reduce pigmentation prior to in situ hybridization, blood staining, and immunocytology (see below). Developing SF and PA-CF larvae (40 hpf) were exposed to a 37 °C heat shock for 1 h^44^. Animals were maintained and handled according to Institutional Animal Care guidelines of the University of Maryland, College Park (IACUC #R-NOV-18-59) (Project 1241065-1).

### Determination of eye size and morphology

Light microscopy and ImageJ software^45^ was used to measure eye and lens areas (Figs. 6m, n and 7d, h) in larvae that were immobilized in 1–2 µg/ml MS222 (Ethyl 3-aminobenzoate methanesulfonate, Western Chemical Inc., Ferndale, WA) or diameters in larvae that were fixed overnight at 4 °C in 4% paraformaldehyde (PFA) following antibody staining (see below). The eye and lens on one side of a head was measured; therefore, the reported *n* values refer to individual larvae, which are biological replicates. To generate the data in Fig. 6j and Supplementary Table 2, eye morphologies were determined in MS222-treated specimens by visual inspection or through ImageJ measurements when appropriate. Specimens were grouped into four categories as follows. Category 1: normal eye (eye approximately the same size as controls), category 2: small eye (eye size determined to be less than controls by visual and/or ImageJ measurements), category 3: category 2-sized eye with lens off center in the retina, and category 4: eye absent. Although categorized on one side of the head, all larvae (except those mentioned as otherwise in the “Results”) expressed the reported morphological categories bilaterally.

### Separation of larval heads and trunks

SF and PA larvae were dissected into heads and trunks by making a single incision at the base of the cranium (Fig. 3a) with a fine steel scalpel blade while they were immersed in a small volume of fish culture system water containing 1 µg/ml MS222 and viewed under the stereomicroscope. The isolated heads and trunks were immediately immersed in Trizol Reagent (Life Technologies, Grand Island, NY), and RNA was extracted.

### cDNA synthesis and RT-PCR

Total RNA was isolated from embryos and larvae using Trizol Reagent, and cDNA was synthesized using the SuperScriptTM III First-Strand Synthesis SuperMix Kit and oligo (dT)_20_ or random hexamer primers (Life Technologies)^17^. Qualitative RT-PCR was done using the PCR Master Kit (Roche Diagnostics GmbH, Mannheim, Germany). The primers used to amplify protein-coding genes from scaffold KB871589.1 in the Ensembl AstMex1.0.2 PA-CF genome release are listed in Supplementary Table 5. The primers used for PCR amplification of other genes with expression levels determined by qualitative RT-PCR are listed in Supplementary Tables 6 and 7. The PCR cycling conditions for qualitative RT-PCR were 1 cycle of initial denaturation at 94 °C for 2 min, followed by 25–32 cycles of denaturation (94 °C for 30 s), annealing (60 °C for 30 s), and elongation (72 °C for 30 s), and a final elongation step at 72 °C for 5 min. The standard was 18S rRNA. Quantitative real-time RT-PCR (qPCR) of *cbsa*, *cbsb*, and hypoxia-sensitive genes was carried using glyceraldehyde 3-phosphate dehydrogenase as a standard^15,45^. The primers used for qPCR are listed in Supplementary Table 7.

### In situ hybridization

RNA probes corresponding to gene-specific sequences in the *cbsa*-, *cryaa*-, *hsf2bp*-, and *cbsb*-coding regions were amplified by PCR from SF cDNA using the primers listed in Supplementary Table 6. The PCR products were cloned into the TOPO vector using the TOPO TA Cloning Kit Dual Promoter (Life Technologies) and confirmed by sequencing. Sense and anti-sense digoxigenin-labeled RNA probes were prepared with SP6 RNA and T7 RNA Polymerases (Roche Diagnostics, Indianapolis, IN). Samples were fixed with 4% PFA, processed for whole-mount in situ hybridization, and photographed using a light microscope^17^.

### F1 hybrid test

F1 hybrid embryos were produced by inseminating SF eggs with PA-CF sperm using in vitro fertilization^45^. RNA was extracted from isolated heads (see above) of SF, PA-CF, and SF X PA-CF F1 hybrids at 60 hpf. Total RNA was extracted, and cDNA was synthesized as described above. The primers for amplification of *cbsa* by RT-PCR were 5’-CGGATGGTGGAAGATGCAGA-3’ (forward) and 5’-CGTAGTGAGCCAGAGGGTTG-3’ (reverse). The PCR cycling conditions were 1 cycle of initial denaturation at 94 °C for 2 min, followed by 28 cycles of denaturation at 94 °C for 30 s, annealing at 60 °C for 30 s, and elongation at 72 °C for 30 s, and a final elongation step at 72 °C for 7 min. Amplification of the control 18S rRNA was carried out using 1 μl of the synthesized cDNA with primers 5’-GAGTATGGTTGCAAAGCTGAAA-3’ (forward) and 5’-CCGGACATCTAAGGGCATCA-3’ (reverse). The PCR cycling conditions were 1 cycle of initial denaturation at 94 °C for 5 min, followed with 25 cycles of denaturation at 94 °C for 30 s, annealing at 62 °C for 30 s, and elongation at 72 °C for 30 s, and a final elongation step at 72 °C for 7 min.

To determine whether the SF or PA-CF *cbsa* allele was expressed in hybrids, PCR reactions were performed to amplify a 327-bp region of the *cbsa*-coding region containing the SF- and CF-specific mononucleotide markers using the forward primer 5’-ACCCTCTGGCTCACTACGAC-3’ and the reverse primer 5’-TGCGAGCCATAGCAAAGGAC-3’ and the Phusion High-Fidelity PCR Master Mix (New England Bio Labs, Ipswich, MA)^17^. The PCR products were purified with the MinElute Gel Extraction Kit (Qiagen, Valencia, CA) and cloned into the Dual Promoter PCR II-TOPO Vector using the TOPO TA Cloning Kit (Life Technologies)^17^. Both PCR products and TOPO plasmids were sequenced. The PCR products were sequenced with forward primer 5’-ACCCTCTGGCTCACTACGAC-3’, and TOPO plasmids were sequenced using the M13-20 and M13rev primers provided with the kit to distinguish which *cbsa* clones contained the SF or CF marker.

### RACE reactions and sequencing

The 5’ and 3’ ends of SF and PA *cbsa* mRNA were determined using the SMARTer™ RACE cDNA Amplification Kit (Clontech Laboratories, Inc., Mountain View, CA). Poly A^+^ RNA was isolated with the NucleoBond RNA/DNA Kit (Macherey-Nagel, Duren, Germany) and RACE-Ready cDNA was generated using the first-strand cDNA synthesis protocol in the SMARTer™ RACE cDNA Amplification Kit (Clontech). The primary gene-specific primer for *cbsa* 5’-RACE was 5’-CGCTCTCTCTGCATCTTCCACCATCCG-3’; and the nested gene-specific primer for *cbsa* 5’-RACE was 5’-GCTGATTCTGTCCTTCACACTGCCGCC-3’; the primary gene specific primer for *cbsa* 3’-RACE was 5’-TCTCTGCTCCCCTCACTGTTTTGCCCA-3’; and the nested gene-specific primer for 3’-RACE was 5’-GGAGACGGATCACTTTGCCCTGGTGGT-3’. The PCR reactions were performed using the Advantage 2 PCR Kit (Clontech). The PCR conditions were 5 cycles at 94 °C for 30 s and 72 °C for 3 min; 5 cycles at 94 °C for 30 s, 70 °C for 30 s, and 72 °C for 3 min; 27 cycles at 94 °C for 30 s, 68 °C for 30 s, and 72 °C for 3 min. For the nested PCR reactions, the cycling conditions were 20 cycles each at 94 °C for 30 s, 68 °C for 30 s, and 72 °C for 3 min. The PCR products were purified with the MinElute PCR Purification Kit (Qiagen) and sequenced with nested gene-specific primers.

### Genome walking and sequencing

The SF and PA *cbsa* gene loci and flanking regions were amplified and sequenced by genome walking^17^. The entire SF and PA-CF *cbsa* genomic DNA sequences were amplified using the GenomeWalker™ Universal Kit (Clontech Laboratories, Mountain View, CA), and the overlapping genomic regions were sequenced step by step using the primers listed in Supplementary Table 8. For the construction of GenomeWalker libraries, genomic DNA was digested with *Eco*RV, *Dra* I, *Pvu*II, and *StuI* I. The GenomeWalker PCR reactions were performed with TaKaRa LA Taq™ (Takara Bio, Mountain View, CA). The PCR reactions were conducted using the two-step cycle parameters described in the GenomeWalker^TM^ Kit manual. After obtaining the major bands, the PCR products were inserted into the TOPO vector, and both the PCR products and TOPO plasmids were sequenced.

To amplify the genomic region between *cbsa* and the adjacent upstream gene *gemin8*, we designed 14 pairs of primers (Supplementary Table 8) using incomplete sequences available in the Ensembl PA-CF database. The PCR reactions were performed by PCR Master (Roche, Roche Diagnostics GmbH, Mannheim, Germany), and the PCR products were inserted into the TOPO vectors, and both the PCR products and TOPO plasmids were sequenced. After sequencing, low-quality reads and pCRII TOPO vector sequences were removed, the sequences were trimmed, and clean genomic sequence reads were assembled with the DNAstar SeqMan Pro software.

To amplify SF and PA-CF *cbsa* intron 6, which was not covered by genome walker generated or Ensembl sequences (AstMex1.0.2), we used the forward primer 5’AGATCGTCCGTACCCCTACC-3’, which corresponds to a region located in *cbsa* exon 5, and the reverse primer 5’-TGCCAGCTGAAGTGTGCTTA-3’, which corresponds to a region located in intron 11. The PCR reactions were performed using Phusion High-Fidelity PCR Master Mix (New England Biolabs). The PCR cycling conditions were 1 cycle of initial denaturation at 98 °C for 30 s, followed by 35 cycles each of denaturation at 98 °C for 8 s, annealing at 68 °C for 25 s, extension at 72 °C for 5.5 min, and a final elongation step at 72 °C for 10 min. The PCR products were purified with the MinElute Gel Extraction Kit (Qiagen) and sequenced.

### Indel sequencing in CF populations

Genomic DNA was extracted from tailfin clips of six different CF populations (see above) using the DNeasy Blood & Tissue Kit (Qiagen). The primers used to amplify the region surrounding the indel in *cbsa* intron 1 were 5’-GCCTGCATGTGCCAGAGGGG-3’ (forward) and 5’-CCGCCGCCAAAACATTGCGT-3’ (reverse). The PCR cycling conditions were 1 cycle of initial denaturation at 98 °C for 30 s, followed by 35 cycles of denaturation (98 °C for 5 s), annealing (at 60 °C for 15 s), extension (at 72 °C for 15 s), and a final extension at 72 °C for 7 min. The primers used to amplify the region surrounding intron 6 were 5’-CCAGAGGCAGACATGTTTCCGATT-3’ (forward) and 5’-GGAGGCTGCAGAGTACTGACAGT-3’ (reverse), and the PCR cycling conditions were the same as those used for the intron 1 region. The primers used to amplify the region surrounding intron 8 were 5’-TGGCTTCAAGCAAGGGCGGG-3’ (forward) and 5’-AGTTGCGGGCAACATCATACCCT-3’ (reverse). The PCR cycling conditions were the same as those used to amplify intron 1, except that annealing was done at 58 °C for 15 s. All PCR reactions were performed using Phusion High-Fidelity PCR Master Mix (New England Biolabs, Ipswich, MA, USA), and the PCR products were purified with the MinElute PCR Purification Kit (Qiagen) and sequenced.

### Enhancer identification and transgenesis

Potential enhancers were identified in *cbsa* non-coding DNA sequence using iEnhancer-2L^21^. The predicted enhancer (E in Fig. 3b) in *cbsa* intron 1, which showed sequence differences between SF and all 6 types of CF, was inserted into the pCMV-GFP vector for expression analysis. The 354-bp region including enhancer E was amplified from SF and PA-CF genomic DNA and inserted into the *Spe*I site of the pCMV-GFP vector. The pSF-E-CMV-CFP and pCF-E-CMV-GFP constructs were confirmed by sequencing. The DNA constructs and transposase mRNA (25 ng each) were injected into 1-cell SF embryos, and GFP expression was determined by fluorescence microscopy at 40 hpf. Predicted transcription factor-binding sites in the *cbsa* intron 1 regions containing indels were identified using the online PROMO (Version 3.0.2) software^46,47^.

### Gene knockdown and rescue by mRNA injection

Knockdown of the *cbsa* and *cbsb* genes was done using the splice inhibiting MOs *cbsa* MO 5’-CAATGCTAATGCTTTTACCTTCTCC-3’, which targeted exon 5 of the *cbsa* gene, and *cbsb* MO 5’-AGCCACTGCAAACACACATACATCA-3’, which targeted *cbsb* exon 4. The control MO was 5’-CCTCTTACCTCAGTTACAATTTATA-3’. The MOs were designed and synthesized by Gene Tools, Inc. (Philomath, OR). The *cbsa* and *cbsb* MOs were injected into 1–2-cell SF embryos at concentrations of 0.25 mM, and the morphants were cultured through the early larval stages.

For RT-PCR validations, the *cbsa* and *cbsb* morphants were collected at 40 hpf and total RNA was isolated and cDNA synthesized as described above. The primers used to amplify the region containing the *cbsa* sequence change were 5’-CGGATGGTGGAAGATGCAGA-3’ (forward) and 5’-CGTAGTGAGCCAGAGGGTTG-3’ (reverse). The primers used to amplify the region containing the *cbsb* sequence change were 5’-GGAAAATTGGAGACACGCCG-3’ (forward) and 5’-ATGATGCAGCGGTAACCCTT-3’ (reverse).

To prepare *cbsa* mRNA for rescue experiments, the full-length coding region was amplified from SF *cbsa* cDNA using 5’-GGGCTCGAGCGAATCAGCACCACCTGAAC-3’ (forward) and 5’-GGGTCTAGAAACCATTCCTTTCAGAGACTGGA-3’(reverse) primers, which included *Xho*I and *Xba*I sites. The purified PCR product was digested with *Xho*I and *Xba*I, ligated into the pCS2+ vector, and the chimeric plasmid was confirmed by sequencing. After linearization with *Not*I, the capped mRNA was transcribed using the mMESSAGE mMACHINE Sp6 Kit (Life Technologies). After transcription, *cbsa* mRNA was recovered by LiCl precipitation, washed in 70% ethanol, re-suspended in sterile H_2_O, and stored at −20 °C. We injected *cbsa* mRNA into 1–2-cell *cbsa* or *cbsb* morphant SF embryos at 75 ng/μl or into 1-cell PA-CF embryos at 100 ng/µl in sterile water containing 0.05% phenol red^17^.

### CRISPR-Cas9 gene editing

Mutations in the SF *cbsa* and *rap1b* genes were induced by CRISPR-Cas9 editing using two guide RNAs (single guide RNA (sgRNA)) to obtain maximal efficiency^48^. The *cbsa* sgRNAs were designed using ENSEMBL ENSAMXT00000008310: the sequence of the first *cbsa* sgRNA was “AAGUGCGAGUACUUCAACGC,” and the sequence of the second *cbsa* sgRNA was “UGCGAGUACUUCAACGCCGG.” The *rap1b* sgRNAs were designed using ENSEMBL ENSAMXT00000030949.1: the sequence of the first *rap1b* sgRNA was “GGAACAAUUCACAGCCAUGA,” and the sequence of the second *rap1b* sgRNA was “AGGAACAAUUCACAGCCAUG.” We co-injected 50 pg of each sgRNA and 300 pg of Cas9 protein (Cas9 nuclease 2NLS, *S. pyrogenes*, Synthego Corp., Redwood City, CA) into one-cell-stage SF embryos. CRISPR-Cas9-injected and control un-injected embryos from the same clutch were cultured at 25 °C, phenotyped at 3.5 dpf by microscopy, genomic DNA was extracted from injected and control larvae, and nested PCR was used to amplify the edited sites. For *cbsa*-edited larvae, the flanking primers were 5’-GTATGCAGACAGCACTACAGG-3’ (forward) and 5’-ACCTGGACGACTGGAATGTTA-3’ (reverse), and the nested primers were 5’-ATGCAGACAGCACTACAGGC-3’ (forward) and 5’-TCTTCTCAAACCCCTCGTCAC-3’ (reverse). For *rap1b*-edited larvae, the flanking primers were 5’-GCACCCCAAAGGCTTGTTTA-3’ (forward) and 5’-GCTACCTACAAAGGAGGCAGA-3’ (reverse), and the nested primers were 5’-ACCCCTGTCATTATGTGGGC-3’ (forward) and 5’-ATGAATGGATGAGTGGCGAGG-3’ (reverse).

### hCys determination and injection

hCys was quantified in SF, SF *cbs* morphants, PA-CF, and TI-CF larvae using the Homocysteine ELISA Kit (Cell Biolabs Inc., San Diego, CA). Samples containing 50 larvae were homogenized in phosphate-buffered saline (PBS), the homogenates were centrifuged at 10,000 × *g* for 15 min at 4 °C, and the supernatant fraction was collected and assayed immediately according to the instructions of the manufacturer.

L-homocysteine and L-cysteine (both purchased from Sigma Aldrich) were diluted to 1–5 mM in sterile water containing 0.05% phenol red, microinjected into 1–2-cell SF embryos at a final concentration of 5 mM, and the effects on eye development were determined in SF larvae at 4 dpf.

### Microangiography

Microangiography^49^ was performed on SF, PA-CF, or *cbs* morphants mounted in 1.2% low-melt agarose prepared in zebrafish embryo medium^50^. Embryos were injected with Qtracker 655 or 705 vascular labels (ThermoFisher Scientific) in the dorsal aorta around the yolk extension area using a pressure injector (World Precision Instruments, Friedberg, Germany). Injected embryos were immediately observed using an upright Leica TCS SP5 confocal microscope. Vascular leakages were analyzed in and around the head regions. At least three to five injected embryos were examined per dose in each experiment.

### Macrophage and red blood cell staining

Vital staining for macrophages was done by incubating 4–10 dpf PA-CF larvae with 25 μg/ml Neutral Red in fish system tank water for 12 h at room temperature in the dark^51^. Embryos were stained for red blood cells for 15 min with 0.6 mg/ml o-dianisidine (Sigma, St. Louis, MO, USA), 0.01 M sodium acetate (pH 4.5), 0.65% hydrogen peroxide, and 40% (v/v) ethanol in the dark^52^. After staining, the embryos were rinsed in PBS, post-fixed in 4% PFA for 1 h, rinsed in PBS, and stored in 80% glycerol.

### Antibody staining

SF and PA-CF larvae from 1.5 to 12 dpf were fixed in 4% PFA overnight at 4 °C, dehydrated through an increasing series of methanol concentrations to 100% methanol, and stored at −20 °C. The larvae were rehydrated, blocked with superblock solution containing 5% goat serum, and incubated in a 1:100 dilution of rabbit polyclonal anti-HIF1α primary antibody (Catalogue Number 114977, Abcam, Cambridge, UK) for 48 h at 4 °C, rinsed with superblock, then incubated with 1:200 dilution of Alexa Fluor 488 goat anti-rabbit IgG secondary antibody (ThermoFisher, Waltham, MA) overnight at 4 °C, rinsed with PBS, 0.1% Triton X-100, 0.2% bovine serum albumin, and visualized by fluorescence microscopy.

### Lens deletion and apoptosis detection

The lens was removed from the optic cup on one side of SF embryos at about 30 hpf by microdissection using Tungsten needles^53^, and hemorrhaging was assayed by light microscopy from 2 to 8 dpf.

Lens apoptosis was determined by vital staining of SF, PA-CF, and *cbs* morphants at 40 hpf with 5 μg/ml Lysotracker Red DND 99 (Invitrogen, Carlsberg, CA) for 30 min in the dark^45^. Stained larvae were anesthetized in MS222 (see above) and mounted on glass slides for imaging.

### Reporting summary

Further information on research design is available in the Nature Research Reporting Summary linked to this article.

## Supplementary information

Supplementary Information

Reporting Summary

## Data Availability

The authors declare that all data supporting the findings of this study are available within the article and its Supplementary Information files or from the corresponding author upon reasonable request. Data are reported in figures, Supplementary Figures, and Supplementary Tables, and raw sequence data have been deposited in the NCBI database under accession codes: MK801789 (SF *cbsa* mRNA), MK801790 (PA-CF *cbsa* mRNA), MN186089 (SF *cbsa* genomic region), and MN186090 (PA-CF *cbsa* genomic region). The source data for Figs. 1, 2, 3c–e, 4b–h, 5, 6k–r, 7, 8, 9, and Supplementary Fig. 4a, b, g, h are provided as a source data file.

## Source data

Source data
