# Author Correction: A hypomorphic *cystathionine ß-synthase* gene contributes to cavefish eye loss by disrupting optic vasculature

**DOI:** 10.1038/s41467-020-19340-5

**Published:** 2020-10-22

**Authors:** Li Ma, Aniket V. Gore, Daniel Castranova, Janet Shi, Mandy Ng, Kelly A. Tomins, Corine M. van der Weele, Brant M. Weinstein, William R. Jeffery

**Affiliations:** 1 Department of Biology, University of Maryland, College Park, MD USA; 2 Division of Developmental Biology, Eunice Kennedy Shriver National Institute of Child Health and Human Development, NIH, Bethesda, MD USA; 3 Department of Genetics, Stanford University, School of Medicine, Stanford, CA USA

**Keywords:** Developmental biology, Evolutionary developmental biology

Correction to: *Nature Communications* 10.1038/s41467-020-16497-x, published online 2 June 2020.

The original version of the Supplementary Information associated with this Article contained an error in Supplementary Fig. 4b, in which both panels of Supplementary Fig. 4a were duplicated in error in Supplementary Fig. 4b. The correct version of Supplementary Fig. 4b is given with this paper, which replaces the previous incorrect version.

The HTML has been updated to include a corrected version of the Supplementary Information.

