# Supplementary material for: A hypomorphic cystathionine ß-synthase gene contributes to cavefish eye loss by disrupting optic vasculature
Source: Nat Commun. 2020 Jun 2;11:2772. doi: 10.1038/s41467-020-16497-x (PMC7265459; doi:10.1038/s41467-020-16497-x)
Supplement: Supplementary file 1 — Supplementary Information [file 41467_2020_16497_MOESM1_ESM.pdf]

A Hypomorphic *Cystathionine ß-Synthase* Gene Contributes to Cavefish Eye Loss by Disrupting Optic Vasculature

Ma et al.

Supplementary Figures and Legends

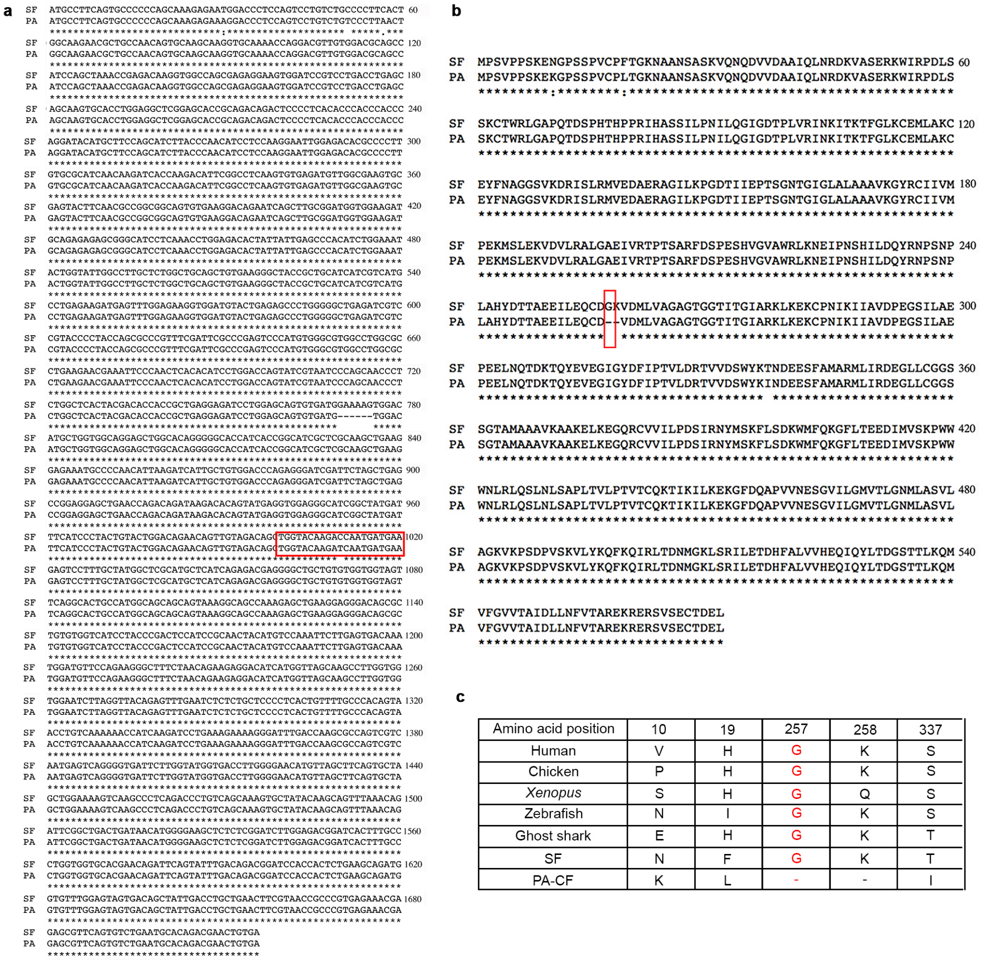

c

Amino acid position	10	19	257	258	337
Human	V	H	G	K	S
Chicken	P	H	G	K	S
Xenopus	S	H	G	Q	S
Zebrafish	N	I	G	K	S
Ghost shark	E	H	G	K	T
SF	N	F	G	K	T
PA-CF	K	L	-	-	I

Supplementary Fig. 1: Nucleotide and protein sequence of the *cbsa* genes. (**a**) Nucleotide coding sequence alignments of the SF and PA-CF *cbsa* genes. Asterisks: identical nucleotides. Spaces, or - - - show different nucleotides or deletions respectively. The region complementary to the sequence used to identify the SF and PA-CF alleles in the F1 hybrid test, including the SNP marker (Fig. 3a), is boxed in red. (**b**) Deduced amino acid sequence alignment of the SF and PA-CF CBSA proteins. Asterisks: identical amino acids. or - - - different amino acids or deletions respectively. The amino acid that is conserved in vertebrates and SF but absent in PA-CF CBSA (G-257) is boxed in red. (**c**) Comparison of divergent amino acid sites in SF and PA-CF CBSA to conserved amino acids at the same position in zebrafish CBSA or other vertebrate CBS proteins. Variable amino acid sites are indicated in black type. The conserved amino acid site (G-257) is indicated in red. Dashes indicate missing amino acids in PA-CF CBSA.

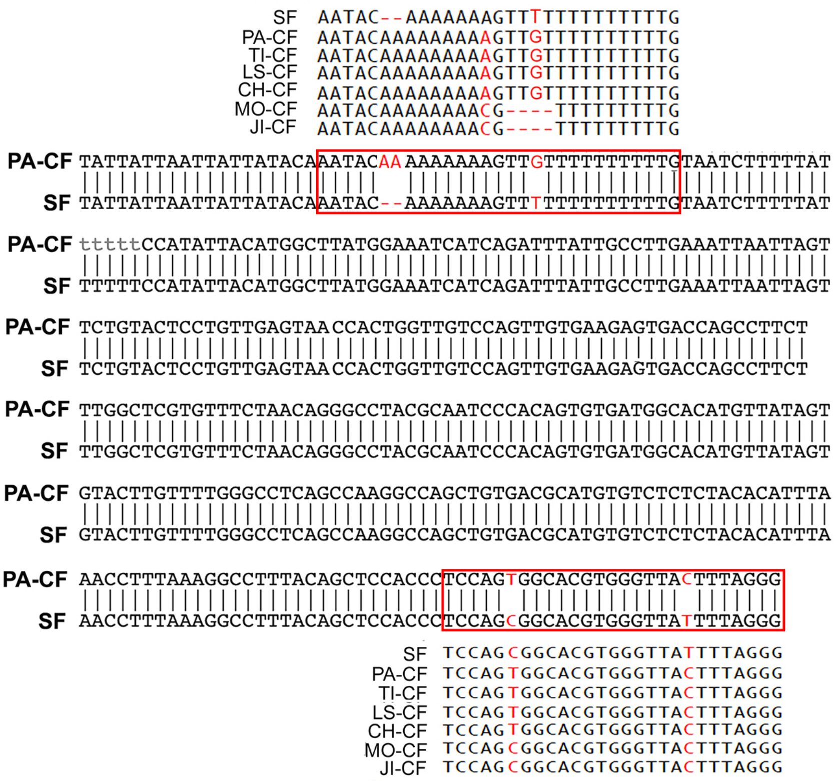

Supplementary Fig. 2: Indels in the E Region of the Cavefish *cbsa* gene. Alignment of the E region of SF and PA-CF and SF showing indels. Boxed regions and aligned sequences above or below: indels (red) shared in six different CF populations.

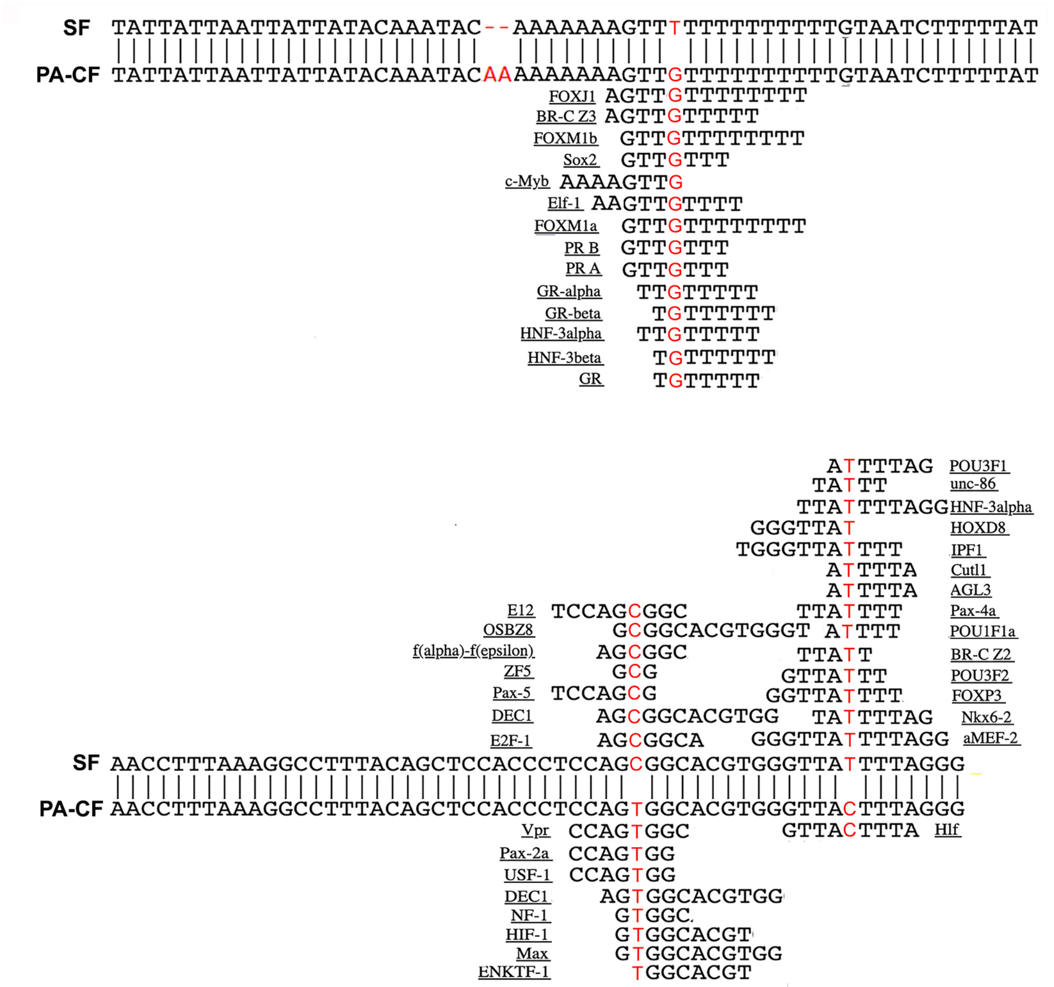

Supplementary Fig. 3: **Predicted transcription factor binding sites in the E region.** Predicted transcription factor binding sites specific for SF or PA-CF indels in 5’ (top) and 3’ (bottom) parts of the E region of the *cbsa* genes. Predicted transcription factors and binding sites specific for SF sequences are shown on top of the alignments and for the mutated PA-CF sequences at the bottom of the alignments. Predicted transcription factors that are unchanged between SF and PA-CF are not shown. Critical indels resulting in changed transcription factor binding sites are indicated in red.

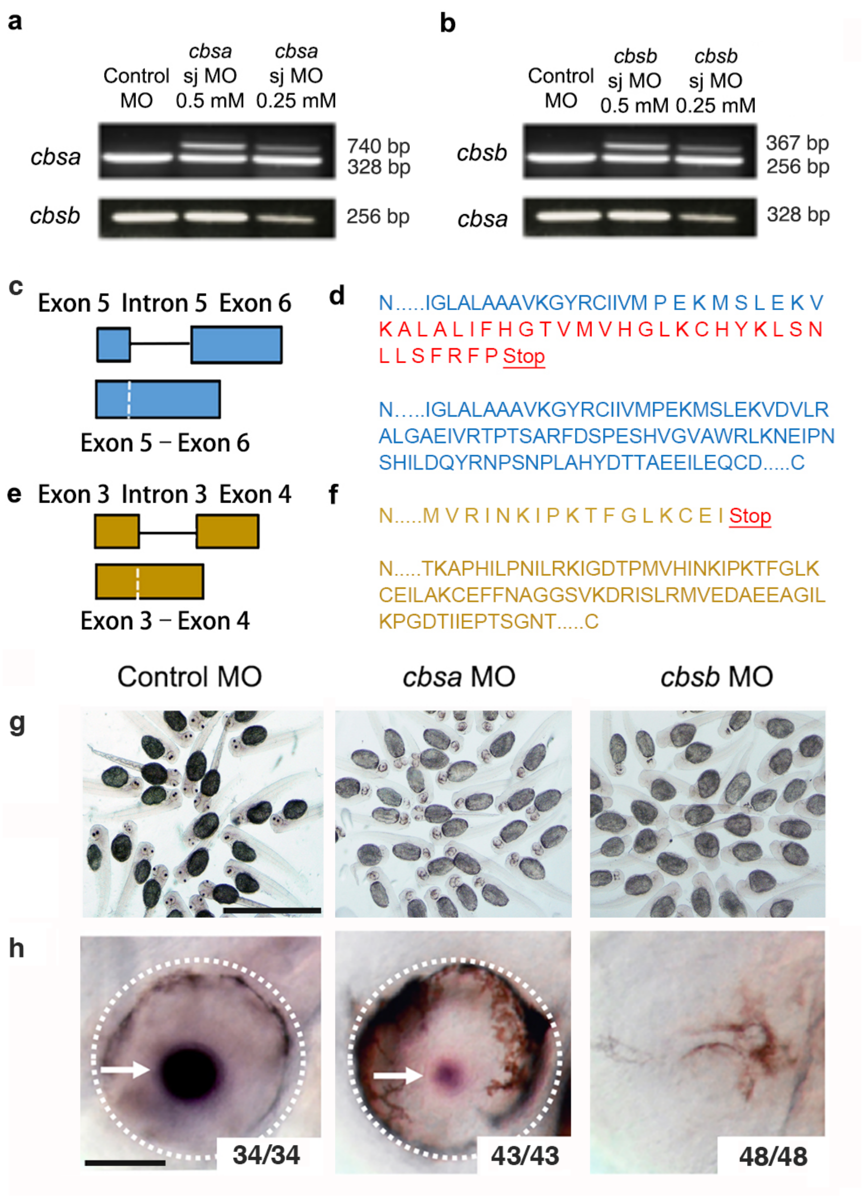

Supplementary Fig. 4: Controls for morpholino based *cbs* gene knockdown. (**a-e**) Validations by RT-PCR showing *cbsa* and *cbsb* expression in morphants produced by injection of 0.5 mM or 0.25 mM *cbsa* (top) or *cbsb* (bottom) MOs into SF eggs. The upper bands in the *cbsa* and *cbsb* MO lanes are PCR products from pre-mRNAs containing unprocessed *cbsa* or *cbsa* introns including translation stop sites (see d, f below). The lower bands are PCR products from processed mRNAs. (**c, e**) Diagrams of the *cbsa* (top, blue) and *cbsb* (bottom, yellow) intron-exon boundary regions (top) and processed exons (bottom) targeted by *cbsa* and *cbsb* splice junction MOs. (**d, f**) Deduced amino acid sequences of *cbs* of sequenced upper and lower PCR products in **a** and **b**. The deduced amino acid sequences of PCR products from the *cbsa* and *cbsb* upper transcripts both contain unprocessed stop codons in introns that would lead to premature translation termination. *cbsa* exons: blue letters. *cbsb* exons: yellow letters.

Introns and translation termination (stop) sites: red letters. (**g, h**). Control, *cbsa*, and *cbsb* morphants (0.5 mM) *in situ* hybridized with a *cryaa* probe at 40 hpf to detect the lens (when present). Upper rows: small groups of *cryaa* stained morphants. Scale bar in g is 1.25 mm; magnification is the same across the upper row. Bottom row: single typical *cryaa* stained morphants. Dashed outlines (eyes). Arrows: lens. Control morphants show normal eyes with *cryaa* stained lenses, *cbsa* morphants showing mostly abnormal eyes with reduced *cryaa* stained lenses, and *cbsb* morphants mostly lacking eyes and *cryaa* stained lenses. Insets: The n of indicated result/total number for g and h. Scale bar in h: 100 μm; magnification is the same across the bottom row. Source data are provided as a source data file.

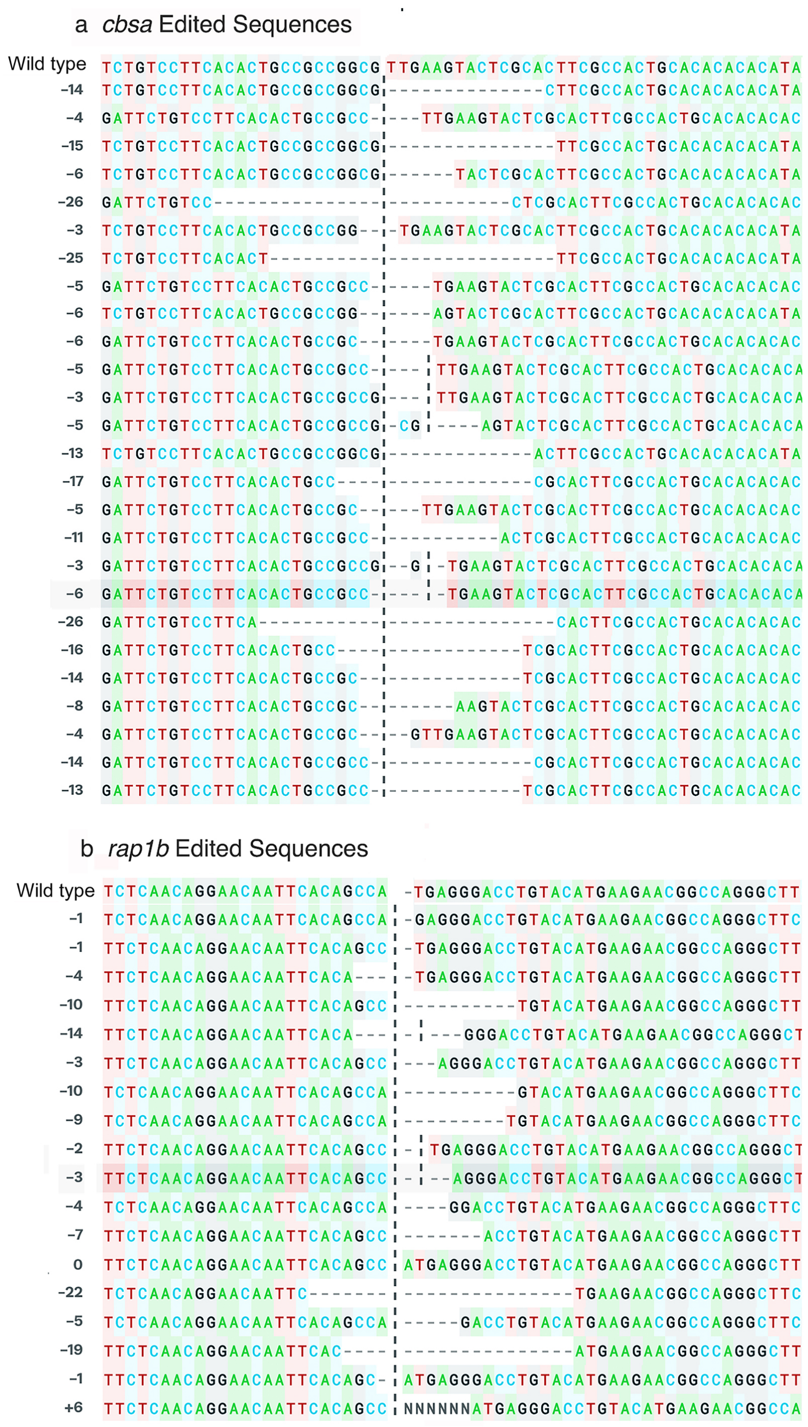

Supplementary Fig. 5: Summary of *cbsa* (a) and *rap1b* (b) CRISPR/Cas9 mutations. The number of deleted nucleotides in each mutation is shown on the left. Dashed vertical lines indicate the beginning of the guide RNAs. Sequences are shown from 5' (left) to 3' (right).

Supplementary Tables

Supplementary Table 1. The presence (+) or absence (-) of indels in *cbsa* introns 6 and 8 in different CF populations compared to SF.

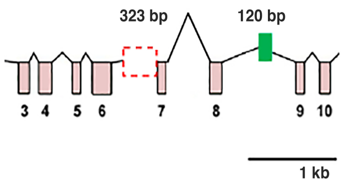

Cavefish Type	Intron 6 323 bp deletion indel	Intron 8 120 bp insertion indel
PA-CF	+	+
TI-CF	+	+
LS-CF	+	+
CH-CF	+	+
MO-CF	-	-
JI-CF	-	-

Supplementary Table 2. Effects of homocysteine (hCys) or cysteine (Cys) injection into SF eggs on eye morphology at 40 hpf. See Materials and Methods for description of morphological categories.

Egg injection	Total number	Number normal eyes	Number small or absent eyes	Percent small or absent eyes (%)
Cys	28	27	1	3.57
hCys	166	128	38	22.89

Supplementary Table 3. Effects of *cbs* morpholino injection into SF eggs on eye morphology at 40 hpf. See Materials and Methods for description of morphological categories.

Morpholino(s)	Total number	Number normal eyes	Percent normal eyes (%)	Number small eyes with ventrally displaced lens	Percent small eyes (%)	Number absent eyes	Percent absent eyes (%)
Control	450	437	97.1	10	2.2	3	0.7
cbsa	907	526	58.0	357	39.4	24	2.6
cbsb	235	7	3.0	35	14.9	193	82.1
cbsa+cbsb	61	2	3.3	5	8.2	54	88.5

Supplementary Table 4. Effects of *cbs* morpholino and *cbsa* mRNA injection into SF eggs on eye morphology at 40 hpf. See Materials and Methods for description of morphological categories.

Injection	Total number	Number normal eyes	Percent normal eyes (%)	Number small eyes with ventrally displaced lens	Percent small eyes (%)	Number of absent eyes	Percent of absent eyes (%)
cbsa MO	28	8	28.6	16	57.1	4	14.3
cbsa MO + *cbsa* mRNA	40	34	85.0	4	10.0	2	5.0
cbsb MO	48	2	4.2	10	20.8	36	75.0
cbsb MO + *cbsa* mRNA	45	0	0	32	71.1	13	28.9

Supplementary Table 5. Gene models and ID numbers of protein coding genes on scaffold KB871589.1 in the Ensembl AstMex102 genome assembly and primers used for screening for gene expression levels in SF and PA-CF at 40 hpf by qualitative RT-PCR.

Gene name	Ensembl version gene ID	Forward primer (5'-3')	Reverse primer (5'-3')
arhgap6-2	ENSAMXG00000007866	TCACCATCCCCAAAGATGGC	GGAGTCTTTGTGCTCCTCCC
arhgap6-3	ENSAMXG00000007844	TTCATGACGTGGCTGCGTT-	AGATCCTCGAACGTGGCGAT
msl3-201	ENSAMXG00000007892	GTTCCCCACTGTTTTGAGCG	TTGCGTGCCAGTTTACGTTG
frmpd4	ENSAMXG00000007924	GACTCACCAAGGAGTGCGAA	GGAGCCAGAGAGGGTAAGGA
pwp2h	ENSAMXG00000007988	CCTGTTCACAGCGTCTCCTT	TCCGTCAGGAGAATAGGCCA
tmsb4x	ENSAMXG00000008001	GAGGTCACCAGCTTCGACAA	ATGACGCTTGCTTCTCCTGT
tceanc	ENSAMXG00000025888	AGCTGCAGACCACTGACATC	CACAGACATGCGGGCAAAAA
rab9a	ENSAMXG00000025889	GGACGGCTGGTCACTTTACA	CCACCTTTGCGCTTCCTCTA
gpm6ba	ENSAMXG00000008016	ATGTGGGCAAAACTTGGCTG	GCGTCGTCCTTGGTCTTGAT
gemin8	ENSAMXG00000008029	CTGTCTATGCCCGCTACTGG	GTCTGTGCAAAGAACTGCCG
cbsa	ENSAMXG00000008046	CGCATGCTCATCAGAGACGA	GGCAAAGTGATCCGTCTCCA
cryaa	ENSAMXG00000008095	TTTGACTATGACCTCTTCCCCTACGC	GGGGGTAGAGTTAGTCTTGTCGTCAC
hsf2bp	ENSAMXG00000008124	AGGAGCAGAAGAAGCAGCAG	ATAAGCGGTATGAGTGCGGG
ankrd10	ENSAMXG00000008136	AACGCAAATGGGTTGACTGC	TCTCCGCATGATCAGGTTCG
ing1	ENSAMXG00000008150	CCTGAGGGGACTCCCTTTGA	AGCTGGACTGTTTGGGTTCG
cars2	ENSAMXG00000008172	CCTGGTACAGTTGTGGACCC	CCCCATGGAGACTCCCAGTA
rab20	ENSAMXG00000008185	CCTGCGCCCAGAAAAATGAG	ATGGAACTGCTCACGACCTG
irs2	ENSAMXG00000025890	GCTCTGTCTTAGGATCGCCC	CCTTGGTGTTTGTTGGAGCG
lig4	ENSAMXG00000008192	TCGCCCTCACAGCGTATTTT	CGTTGAGGGCGTTGACTACT
fam155a	ENSAMXG00000008195	TATACACGGAGGCACTCCCA	GGGAGATGGGTTTTGGAGCA

Supplementary Table 6. Oligonucleotide primers used to prepare probes for *in situ* hybridization (ISH) or to amplify *atf3* and *shha* RNA by qualitative RT-PCR.

Transcript	Forward primer (5'-3')	Reverse primer (5'-3')
cbsa (ISH)	CGCATGCTCATCAGAGACGA	GGCAAAGTGATCCGTCTCCA
hsf2bp (ISH)	AGGAGCAGAAGAAGCAGCAG	ATAAGCGGTATGAGTGCGGG
cbsb (ISH)	CGATGGTGCGCATCAACAAA	TGACGATGATGCAGCGGTAA
cryaa (ISH)	TTTGACTATGACCTCTTCCCCT ACGC	GGGGGTAGAGTTAGTCTTGTCG TCAC
shha (RT-PCR)	TATGAAGGCCGGGCCGTGGA	CCGGGTACGACGTTGCTCGC
atf3 (RT-PCR)	ACGCTCGACGACTTTACCAC	GGTGCTCTCCTTGATCTGCT

Supplementary Table 7. Oligonucleotide primers used in quantitative real-time RT-PCR determinations.

Gene name	Ensembl version gene ID	Forward primer (5'-3')	Reverse primer (5'-3')
rps3a	ENSAMXG00000021691	TGTTCAACATCCGC AACCTG	CGGAGGCGATTTTA GTTCCC
hif1αa	ENSAMXG00000039550	GCACTTTACCTACTG CGACG	TGGCAAACAAGTTG TGGTGA
hif1αb	ENSAMXG00000019342	TACAACAGGGATGT CTGCGG	TGGGCATTCTGGAT GGCTAA
hpx	ENSAMXG00000002129	TATGCTTTCAGAGG CCACCA	TAGGAGAAGACAGC GTCCAC
mb	ENSAMXG00000030396	AAAGTTGGGAATCG GTCGGA	CCATGGCCTCGGAT GAGTT
osgn1	ENSAMXG00000037872	CCGAACCCGAACAC CCA	TCCATGACTGAAGC TCGGG
cbsa	ENSAMXG00000008046	ACAGATTCGGCTGA CTGATAAC	GAGAAGAGGTGTGC TCAAACT
cbsb	ENSAMXG00000018461	CTGGAGCAGTGTGA TGGTAAA	GGGTCCACTCCAAC AATCTT
GAPDH	ENSAMXG00000039361	TCCTGAACTCAATG GCAAGC	TTCTCCAAGCGGAC AGTCAA

Supplementary Table 8. Primers used for PCR amplification and genome walking in the SF and PA-CF *cbsa* gene loci.

Primer	Primer sequence 5’-3’
Ma171	GTTATGCCTGAGAAAATGAG-
Ma172	AAGAACTTAGACATGTAGTT
Ma173	AAAGTGGATATGCTGGTGGC
Ma174	AAGTTGAGAAGATCAATGGC
Ma175	ACGGATCCACCACTCTGAAGCA
Ma176	GCTCTCGTTTCTCACGGGCGG
Ma177	TTTGACAGACGGATCCACCACTCT
Ma178	ACGCTCTCGTTTCTCACGGGC
Ma305	GAGCAGTGTGATGGTTGTGG
Ma306	CGGGTAGGATGACCACACAG
Ma307	CGCATGCTCATCAGAGACGA
Ma308	GGCAAAGTGATCCGTCTCCA
Ma329	CGCTCTCTCTGCATCTTCCACCATCCG
Ma330	GCTGATTCTGTCCTTCACACTGCCGCC
Ma331	TCTCTGCTCCCCTCACTGTTTTGCCCA
Ma332	GGAGACGGATCACTTTGCCCTGGTGGT
Ma339	TCACCAAGACATTCGGCCTC
Ma340	AAGCAGTGGTATCAACGCAG
Ma341	TCGTTGTAAATCGACGGCCA
Ma342	CACAGTTGGTGTTGTTTTTGAGC
Ma343	CAGCCACCCAGATAAAGCGA
Ma344	AAGCAGTGGTATCAACGCAGA
Ma345	TGGGAGAAGAGAAAACAGCGG
Ma346	ACAGTTGGTGTTGTTTTTGAGCTT
Ma347	CGGCAGTGTGAAGGACAGAA
Ma348	GAACGCTCTCGTTTCTCACG
Ma349	AGTGCGAGTACTTCAACGCC
Ma350	AGACACTGAACGCTCTCGTTT
Ma351	ACGGCCATGTGTGTTTGTTG
Ma352	GAGGCCGAATGTCTTGGTGA
Ma353	CATAATGGCCTGGGGGATAGC
Ma354	GAAGTACTCGCACTTCGCCA
Ma405	TGTCAGTGAACAATGTTGATGTT
Ma406	GGATCAAATACTTCTTGGACTCAC
Ma407	CAGACAGTAGTGTACAGATTCCCA
Ma408	CACCGGTTGTTCAGGTCCAT
Ma409	AGTAGTGTACAGATTCCCATTGC
Ma410	TGCATGGATGGGGTGTTTGT
Ma437	CGGATGGTGGAAGATGCAGA

Ma438	CGTAGTGAGCCAGAGGGTTG
Ma439	CAGAATCAGCTTGCGGATGG
Ma440	CTGGTAGGGGTACGGACGAT
Ma441	CGATGGTGCGCATCAACAAA
Ma442	TGACGATGATGCAGCGGTAA
Ma443	GGAAAATTGGAGACACGCCG
Ma444	ATGATGCAGCGGTAACCCTT
Ma445	AATCAGCACCACCTGAACCT
Ma446	CAGCCACCGCGAAAATGAC
Ma447	GGATATGCTGCAAGCCGAGA
Ma448	CGGGGTAAAGTTCACGGGAG
Ma449	GCCGAGAAAATGGGTAAAGCC
Ma450	TCCGTTATCGGGGAAGAAGG
Ma451	TCACCAAGACATTCGGCCTC
Ma452	TGTAAAACGACGGCCAGTGA
Ma453	CAGCCACCCAGATAAAGCGA
Ma454	TTGGGTAACGCCAGGGTTTT
Ma481	TGGGTGGGTGGGTGTGAGGGGAGTCTGT
Ma482	GGTGCTCCGAGCCTCCAGGTGCACTT
Ma483	TCTCGCTGGCCACCTTGTCTCGGTTTA
Ma484	TCCTGTCTGCCCCTTCACTGGCAAGAA
Ma485	CTGCCAACAGTGCAAGCAAGGTGCAAAA
Ma486	CGTTGTGGACGCAGCCATCCAGCTAAA
Ma487	TCACCAAGACATTCGGCCTC
Ma488	CCATGGCAGTGCCTGAACTA
Ma489	AGATCGTCCGTACCCCTACC
Ma490	TGCCAGCTGAAGTGTGCTTA
Ma491	TGGGGTTTGGTGCTCGTATG
Ma492	AGACAGCCTTGCACCTACAG
Ma 493	GGTCCTAAAGCCTGTTGGGT
Ma 494	TGTTGGTCGTTCGTCCAATTAT
Ma 495	TGGACGAACGACCAACATTT
Ma 496	TGGCACAACCCATATTTGCAT
Ma 497	AATTGGACGAACGACCAACA
Ma 498	CTTAGCTGCATATCATTGAA
Ma 499	AGAACTCTGACTCAGTTGTACATT
Ma 500	AGACACTTAATAGGACCACAAACA
Ma 501	GCCAGTCTGATCTGGTGCTT
Ma 502	TGGCGGTTACTTATCATGTGT
Ma 503	TGTAAGAAGAGCAACATTACGGT
Ma 504	ATCTATCCTGCCCCTTGCAC
Ma 505	GCCTGCTGGACCACTCAAA
Ma 506	GGATCTGAATGATGTCTGTT
Ma 507	GGTGAAGTGATGGACCTGTT
Ma 508	GGACTATACCTATTATACT

Ma 509	GGGAGCTTTTTAACTGTCAG
Ma 510	CTACTGAACATTAGTGGTGCA
Ma 511	ACTGTTCTGTCCAGTACAG
Ma 512	CGATCAGCATCACTTTACTT
Ma 513	CCTGTTAGCCCATATCAGAT
Ma 514	TGACGCACTGATCTACGTGA
Ma 515	CCTCGATTAGCATATTGTCA
Ma516	TGCCGCCGGCGTTGAAGTACTCGCACTT
Ma517	GTTGATGCGCACAAGGGGCGTGTCTCCAA
Ma518	GGCACGTGGGGTTTGGTGCTCGTATGT
Ma519	ACAGCGCGTTCCCACACGTGGCTCGTT
Ma520	TGGCTGCCTTTACTGCTGCTGCCATGGC
Ma521	ACCACACAGCAGCCCCTCGTCTCTGATG
Ma522	GGGTGATTCTTGGTATGGTGACCTTGG
Ma523	ATGGGGAAGCTCTCTCGGATCTTGGAG
Ma524	TGCTCAAGTGTGCTTGTCAG
