# Supplementary material for: A hypomorphic cystathionine ß-synthase gene contributes to cavefish eye loss by disrupting optic vasculature
Source: Nat Commun. 2020 Jun 2;11:2772. doi: 10.1038/s41467-020-16497-x (PMC7265459; doi:10.1038/s41467-020-16497-x)
Supplement: Supplementary file 2 — Reporting Summary [file 41467_2020_16497_MOESM2_ESM.pdf]

nature research

Corresponding author(s): William R. Jeffery

Last updated by author(s): April 12 2020

Reporting Summary

Nature Research wishes to improve the reproducibility of the work that we publish. This form provides structure for consistency and transparency in reporting. For further information on Nature Research policies, see Authors & Referees and the Editorial Policy Checklist .

Statistics

For all statistical analyses, confirm that the following items are present in the figure legend, table legend, main text, or Methods section.

n/a	Confirmed	
☐	☒	The exact sample size (n) for each experimental group/condition, given as a discrete number and unit of measurement
☐	☒	A statement on whether measurements were taken from distinct samples or whether the same sample was measured repeatedly
☐	☒	The statistical test(s) used AND whether they are one- or two-sided<br>*Only common tests should be described solely by name; describe more complex techniques in the Methods section.*
☒	☐	A description of all covariates tested
☒	☐	A description of any assumptions or corrections, such as tests of normality and adjustment for multiple comparisons
☐	☒	A full description of the statistical parameters including central tendency (e.g. means) or other basic estimates (e.g. regression coefficient) AND variation (e.g. standard deviation) or associated estimates of uncertainty (e.g. confidence intervals)
☐	☒	For null hypothesis testing, the test statistic (e.g. F, t, r) with confidence intervals, effect sizes, degrees of freedom and P value noted<br>*Give P values as exact values whenever suitable.*
☒	☐	For Bayesian analysis, information on the choice of priors and Markov chain Monte Carlo settings
☒	☐	For hierarchical and complex designs, identification of the appropriate level for tests and full reporting of outcomes
☒	☐	Estimates of effect sizes (e.g. Cohen's d, Pearson's r), indicating how they were calculated

Our web collection on statistics for biologists contains articles on many of the points above.

Software and code

Policy information about availability of computer code

Data collection	Data are reported in figures, supplementary figures, and supplementary tables, and sequence information has been deposited in NCBI as GenBank accession numbers MK801789 (SF cbsa mRNA), MK801790 (PA-CF cbsa mRNA), MN186089 (SF cbsa genomic region), and MN186090 (PA-CF cbsa genomic region).
Data analysis	Potential enhancers were identified in cbsa non-coding DNA sequence using iEnhancer-2L (http://bioinformatics.hitsz.edu.cn/iEnhancer-2L/). The cbsa CRISPR targets were designed with the online tool https://www.synthego.com/products/bioinformatics/crispr-design-tool and the sequence results were confirmed online http://ice.synthego.com/

For manuscripts utilizing custom algorithms or software that are central to the research but not yet described in published literature, software must be made available to editors/reviewers. We strongly encourage code deposition in a community repository (e.g. GitHub). See the Nature Research guidelines for submitting code & software for further information.

Data

Policy information about availability of data

All manuscripts must include a data availability statement. This statement should provide the following information, where applicable:

- Accession codes, unique identifiers, or web links for publicly available datasets
- A list of figures that have associated raw data
- A description of any restrictions on data availability

Data are reported in figures, supplementary figures, and supplementary tables, and sequence information has been deposited in NCBI as GenBank accession numbers MK801789 (SF cbsa mRNA), MK801790 (PA-CF cbsa mRNA), MN186089 (SF cbsa genomic region), and MN186090 (PA-CF cbsa genomic region). The source data underlying Figs 1, 2, 3c-e, 4b-h, 5, 6k-r, 7, 8, 9 and Supplementary Fig 4a, b, g, h are provided as a Source Data file.

Field-specific reporting

Please select the one below that is the best fit for your research. If you are not sure, read the appropriate sections before making your selection.

- [x] Life sciences
- [ ] Behavioural & social sciences
- [ ] Ecological, evolutionary & environmental sciences

For a reference copy of the document with all sections, see nature.com/documents/nr-reporting-summary-flat.pdf

Life sciences study design

All studies must disclose on these points even when the disclosure is negative.

Sample size	Sample sizes were chosen to reflect standard norms in data collection in particular experiments
Data exclusions	No data were excluded from the analyses
Replication	At least three biological replicates, and usually more, were evaluated in each experiment.
Randomization	Randomization was not relevant to this study because extremely large samples sizes were not a part of the analyses
Blinding	Blinding was not possible, as relevant data analysis was done by microscopy

Reporting for specific materials, systems and methods

We require information from authors about some types of materials, experimental systems and methods used in many studies. Here, indicate whether each material, system or method listed is relevant to your study. If you are not sure if a list item applies to your research, read the appropriate section before selecting a response.

Materials & experimental systems

n/a	Involved in the study	
[ ]	[x]	Antibodies
[x]	[ ]	Eukaryotic cell lines
[x]	[ ]	Palaeontology
[ ]	[x]	Animals and other organisms
[x]	[ ]	Human research participants
[x]	[ ]	Clinical data

Methods

n/a	Involved in the study	
[x]	[ ]	ChIP-seq
[x]	[ ]	Flow cytometry
[x]	[ ]	MRI-based neuroimaging

Antibodies

Antibodies used	Homocycsteine ELISA Kit, and Rabbit polyclonal anti-HIF1α primary antibody (Catalogue Number 114977, Abcam, Cambridge, UK) was used in this study.
Validation	Validation statements on the Abcam web site include a statement of predicted reactivity with teleosts (zebrafish). We validated specificty for Astyanax by Western blotting, obtaining a single polypeptide band of molecular mass consistent with HIF1α.

Animals and other organisms

Policy information about studies involving animals; ARRIVE guidelines recommended for reporting animal research

Laboratory animals	Labortory raised animals were Astyanax mexicanus of the following stains: surface fish (Nacimiento del Rio Choy), Pachon, Tinaja, Chica, Los Sabinos, Jineo, and Molino cavefish; adult sexes: males and females; larval sexes cannot be determined.
Wild animals	No wild captured animals were used in this study
Field-collected samples	No field cultureed animals were used in this study
Ethics oversight	Animals were maintained and handled according to Institutional Animal Care guidelines of the University of Maryland , College Park (IACUC #R-NOV-18-59) (Project 1241065-1).

Note that full information on the approval of the study protocol must also be provided in the manuscript.
